# Early-onset and classical forms of type 2 diabetes show impaired expression of genes involved in muscle branched-chain amino acids metabolism

**DOI:** 10.1038/s41598-017-14120-6

**Published:** 2017-10-23

**Authors:** María Isabel Hernández-Alvarez, Angels Díaz-Ramos, María Berdasco, Jeff Cobb, Evarist Planet, Diane Cooper, Agnieszka Pazderska, Krzystof Wanic, Declan O’Hanlon, Antonio Gomez, Laura R. de la Ballina, Manel Esteller, Manuel Palacin, Donal J. O’Gorman, John J. Nolan, Antonio Zorzano

**Affiliations:** 1 Institute for Research in Biomedicine (IRB Barcelona). The Barcelona Institute of Science and Technology, Barcelona, Spain; 2 Departament de Bioquímica i Biologia Molecular, Facultat de Biologia, 08028 Barcelona, Spain; 3 CIBER de Diabetes y Enfermedades Metabólicas Asociadas (CIBERDEM), Instituto de Salud Carlos III, Madrid, Spain; 4 Cancer Epigenetics and Biology Program, Bellvitge Biomedical Research Institute (IDIBELL), Barcelona, Spain; 5 Metabolon, Inc., Durham, NC USA; 6 Metabolic Research Unit, St James’s Hospital and Trinity College Dublin, Dublin, Ireland; 7 National Institute for Cellular Biotechnology, 3U Diabetes Partnership & School of Health and Human Performance, Dublin City University, Dublin, Ireland; 8 CIBERER, Instituto de Salud Carlos III, Madrid, Spain; 9 Steno Diabetes Center, Gentofte, Denmark

## Abstract

The molecular mechanisms responsible for the pathophysiological traits of type 2 diabetes are incompletely understood. Here we have performed transcriptomic analysis in skeletal muscle, and plasma metabolomics from subjects with classical and early-onset forms of type 2 diabetes (T2D). Focused studies were also performed in tissues from ob/ob and db/db mice. We document that T2D, both early and late onset, are characterized by reduced muscle expression of genes involved in branched-chain amino acids (BCAA) metabolism. Weighted Co-expression Networks Analysis provided support to idea that the BCAA genes are relevant in the pathophysiology of type 2 diabetes, and that mitochondrial BCAA management is impaired in skeletal muscle from T2D patients. In diabetic mice model we detected alterations in skeletal muscle proteins involved in BCAA metabolism but not in obese mice. Metabolomic analysis revealed increased levels of branched-chain keto acids (BCKA), and BCAA in plasma of T2D patients, which may result from the disruption of muscle BCAA management. Our data support the view that inhibition of genes involved in BCAA handling in skeletal muscle takes place as part of the pathophysiology of type 2 diabetes, and this occurs both in early-onset and in classical type 2 diabetes.

## Introduction

Type 2 diabetes mellitus is associated with alterations in glucose and lipid metabolism in skeletal muscle^1^. Some of those alterations are secondary to insulin resistance, which in turn, has been explained by defects at one or several levels of the insulin signaling cascade^2,3^. Thus, glucose transport or glycogen synthesis are defective, which leads to reduced glucose uptake in muscle in type 2 diabetic patients^4–6^. In addition, type 2 diabetes is associated with mitochondrial dysfunction, and reduced expression of PGC-1alpha^7–10^. There is significant variability in the pathophysiology of type 2 diabetes with varying implications for insulin signalling and energy metabolism in skeletal muscle^11^. In this regard, we have previously reported that early-onset very obese type 2 diabetic patients failed to improve VO_2_max and had no improvement in whole-body insulin sensitivity in response to an exercise intervention^12^. In keeping with the possibility that chronic exercise training failed to activate a mitochondrial oxidative response in young type 2 diabetes, acute exercise did not induce PGC-1alpha gene expression, and chronic exercise did not induce Mfn2 expression in early-onset type 2 diabetic patients^13^.

Branched-chain amino acids (BCAAs), leucine, isoleucine and valine, are essential amino acids. Due to essentiality, rates of tissue utilization are exquisitely regulated in humans. It is also known that obesity and type 2 diabetes show alterations in BCAAs plasma levels^14^. Obese and insulin-resistant individuals show higher serum BCAAs than their lean and insulin-sensitive counterparts^15–17^. In addition, type 2 diabetic patients show higher levels of BCAA compared to nondiabetic subjects^18^. Notably, high plasma levels of BCAAs predict the development of type 2 diabetes^19^. These alterations in systemic BCAAs have been discussed as a consequence of insulin resistance^20^, although the mechanisms remain unexplained^14,21^. Interestingly, it has also been proposed that alterations in BCAA availability or metabolism may influence metabolic homeostasis. Regarding to skeletal muscle. BCAAs and more specifically leucine stimulate mTORC1 activity, also known as mammalian target of rapamycin complex 1, a protein complex that functions as a nutrient sensor and controls cell growth^22–24^. mTORC1 also stimulates protein synthesis and inhibits autophagy^25,26^. It has been recently reported that the BCAA metabolite 3-hydroxyisobutyrate is secreted from muscle and causes insulin resistance^27^. Furthermore, alterations in the metabolism of BCAA in mice heterozygous for the BCAA enzyme methylmalonyl-CoA mutase was linked to impaired lipid metabolism^28^.

In this manuscript we have identified additional pathophysiological traits in classical and in early-onset forms of type 2 diabetes, through the use of transcriptomic analysis in skeletal muscle and plasma metabolomics. We document that those different forms of T2D are characterized by reduced muscle expression of genes involved in branched-chain amino acids (BCAA) handling.

## Materials and Methods

### Study subjects

Patients attending either the endocrinology or diabetes services at St James’ Hospital, Dublin and had obesity or type 2 diabetes, were invited to take part in the study. The study protocol was approved by the local Research Ethics Committee and a consent was obtained from all participants. Funding was provided through DEXLIFE, an EU FP7 project (grant agreement no: 279228). The project was approved by the ‘Joint St. James’s Hospital and Adelaide and Meath Hospital Ethics Committee’ reference number 2008/06/03.

Early onset type 2 diabetes was defined as onset of the disease before 30 years of age. Subjects with co-existing illnesses or secondary forms of diabetes were excluded. Classical onset was defined as disease onset after 50 years of age. All recruited patients were obese (BMI > 30) and weight stable, had negative glutamic acid decarboxylase (GAD) antibodies and normal or high fasting c-peptide concentrations at diagnosis (C-peptide ≥ 2.5 ng/ml). Exclusion criteria included a history of ketosis or ketoacidosis, pregnancy, active cancer and use of corticosteroids. Healthy, obese controls with normal glucose tolerance and no family history of diabetes were recruited from endocrine clinics at St James’s Hospital, and through Dublin City University by local advertisement. All subjects were sedentary at baseline (determined using the International Physical Activity Questionnaire – Long Format), with a low VO_2_max. All gave written informed consent for the study which had been approved by the local Research Ethics Committee. Clinical characteristics of both groups of subjects, is summarized in Table 1. Family history was positive for diabetes in a 1^st^ degree relative in 46% and 39% of patients with early- and classical-onset type 2 diabetes, respectively. The duration of diabetes prior to study enrollment was 2.1 years for early-onset and 4.8 years for classical-onset disease. Patients had stable blood glucose concentrations at enrolment, with 88% of early onset type 2 diabetes subjects, and 78% of classical diabetes subjects treated with oral glucose lowering medications. Insulin was administered by 23% and 17% of early- and classical-onset type 2 diabetes participants, respectively.

**Table 1:** Anthropometric and metabolic parameters measured in control and Type 2 diabetic subjects either Early-onset or classical Type 2 diabetes.

	Early-onset type 2 diabetes		Classical type 2 diabetes	
	Control subjects	Youth with type 2 diabetes	Older control subjects	Older type 2 diabetic subjects
n	12	21	17	24
Male/female ratio	9/3	14/7	7/10	18/6
Age (years)	23.82 ± 5.81	28.38 ± 3.86*	54.5 ± 6.09	55.04 ± 6.51
BMI (kg/m2)	35.66 ± 4.44	35 ± 9.66	33.37 ± 3.79	35.17 ± 4.72
Fasting glucose (mmol/l)	5.04 ± 0.51	9.08 ± 2.82*	5.02 ± 0.68	9.60 ± 2.69*
Fasting insulin (pmol/l)	29.59 ± 44.81	31.29 ± 34.64	12.03 ± 7.41	26.97 ± 22.26*
VO2max (ml*kg-1*min-1)	24.43 ± 4.02	24.17 ± 5.47	20.29 ± 5.03	21.93 ± 4.94

Data are means ± standard deviation. Statistical analyses comparing control and type 2 diabetes state were performed by unpaired *t*-Test.

Studies were done as previously described^12,13^. A maximum oxygen consumption capacity (VO_2_max) test was used to assess aerobic capacity and fitness. The maximal progressive incremental test to exhaustion was performed using a computer controlled electromagnetically braked medical assessment bicycle ergometer (Ergoselect 100, Ergoline). Open-circuit indirect calorimetry (Innocor INN00500, Innovision) was used for breath-by-breath respiratory gas analysis and to determine ventilation through a connected silicone rubber mouthpiece. Subjects were instructed to maintain a cycling cadence of 70 to 80 revolutions per minute, with the workload at level 1 set at 50 Watts and increased by 25 Watts every 3 minutes until volitional exhaustion. Blood pressure and heart rate were measured throughout. The test was considered maximal if two of the following criteria were satisfied: a maximal heart rate equal to or greater than 95% of predicted maximal heart rate (220 - age), a respiratory exchange ratio greater than 1.1, or a levelling off in oxygen consumption in spite of further increases in power output.

### Microarray assays

Microarray services were provided by the IRB Functional Genomics Core Facility, including quality control tests of total RNA by Agilent Bioanalyzer and Nanodrop spectrophotometry. Briefly, cDNA library preparation and amplification were performed from 25 ng total RNA using WTA2 (Sigma-Aldrich) with 17 cycles of amplification. 8 µg cDNA were subsequently fragmented by DNaseI and biotinylated by terminal transferase obtained from GeneChip Mapping 250 K Nsp Assay Kit (Affymetrix). Hybridization mixture was prepared according to Affymetrix protocol. Each sample was hybridized to a PrimeView Human array (Affymetrix). Arrays were washed and stained in a Fluidics Station 450 (Fluidics protocol FS450_002) and scanned in a GeneChip Scanner 3000 (both Affymetrix) according to manufacturer’s recommendations. CEL files were generated from DAT files using GCOS software (Affymetrix). Normalized expression intensities were computed from Affymetrix CEL files using the RMA algorithm^29^. Microarray pre-processing was performed using R package (R Core Team (2014) (see Supplementary Statistical Analysis section).

### Metabolomics

For absolute quantitation, metabolites were analyzed by isotope dilution UHPLC-MS/MS assays. In brief, 50 µl of EDTA plasma was spiked with stable labeled internal standards and subsequently subjected to protein precipitation by mixing with 200 µl of 1% formic acid in methanol. Following centrifugation, aliquots of clear supernatant were injected onto an Agilent 1290/AB Sciex QTrap 5500 mass spectrometer LC-MS/MS system equipped with a turbo ion-spray source using two different chromatographic systems (mobile phase/column combinations). 3-Methyl-2-oxobutyric acid, 3-methyl-2-oxovalerate, and 4-methyl-2-oxopentanoic acid were eluted with a gradient (mobile phase A: 0.01% formic acid in water; mobile phase B: acetonitrile/methanol 1:1) on a Waters Acquity C-18 BEH column (2.1 mm × 100 mm, 1.7 µm particle size) and detected in negative mode. Isoleucine, leucine, and valine were eluted with a gradient (mobile phase A: 0.05% perfluoropentanoic acid in water; mobile phase B: 0.05% perfluoropentanoic acid in acetonitrile) on a Waters Acquity C-18 BEH column (2.1 mm × 100 mm, 1.7 mm particle size) and detected in positive mode. Quantitation was performed based on the area ratios of analyte and internal standard peaks using a weighted linear least squares regression analysis generated from fortified calibration standards in water, prepared immediately prior to each run. Stable isotope labeled compounds 3-Methyl-2-oxobutyric acid -d_7_, 4-methyl-2-oxopentanoic acid -d_3_ (used for both 3-methyl-2-oxovalerate and 4-methy-2-oxopentanoic acid), isoleucine-^13^C_6_, leucine-d_3_, valine-^13^C_5_-^15^N, were used as internal standards.

### Mouse models

All animal work was approved and conducted according to guidelines established. This project has been assessed favourably by the Institutional Animal Care and Use Committee from Parc Cientific de Barcelona (IACUC-PCB) and the IACUC considers that the above-mentioned project complies with standard ethical regulations and meets the requirements of current applicable legislation (RD 53/2013 Council Directive; 2010/63/UE; Order 214/1997/GC).

### Statistical analysis

Differential expression analysis of microarray data was performed using GaGa (Rossell D. (2009). “GaGa: a Parsimonious and Flexible Model for Differential Expression Analysis.” Annals of Applied Statistics, 3, pp. 1035-1051) using a 5% FDR and a minimum 1.2 fold-change between groups. Microarray data were adjusted for batch and gender. Relevant results for differential expression analysis were represented in a heatmap showing expression values centered and scaled across samples. In the heatmap, samples and probesets were represented under an hierarchical clustering in which correlation distance and Ward agglomeration method was used. Gene set enrichment was assessed through Bayesian enrichment (Mallick, B. K., Gold, D. & Baladandayuthapani, V. Bayesan Analysis of Gene Expression Data (Wiley, 2009)). One thousand posterior samples were generated to be used in the differential expression indicator, according to the posterior probabilities of differential expression obtained from pairwise comparisons. For each gene set we computed the probability of enrichment as the proportion of posterior samples for which the percentage of differentially expressed genes in the set was larger than the percentage of differentially expressed genes in the rest of the genome.

To measure the association between gene expression and plasma metabolites, Pearson Correlation Coefficients and their corresponding assymptotic 95% confidence intervals and p-values were computed. Correlation coefficients were presented in a symmetric heatmap after clusterization with euclidean distance and Ward agglomeration method.

## Results

### Clinical and metabolic characteristics

The clinical and metabolic characteristics of the four study groups are presented in Table 1. All groups were obese and were matched for body mass index (BMI). The Old and Young T2 diabetes groups had significantly higher fasting blood glucose. Maximal aerobic capacity (VO_2_ max) was similar within the age categories but was lower overall for the older compared to the younger groups.

### Identification of gene expression signatures that are common in early-onset and classical forms of type 2 diabetes

**Figure 1:**
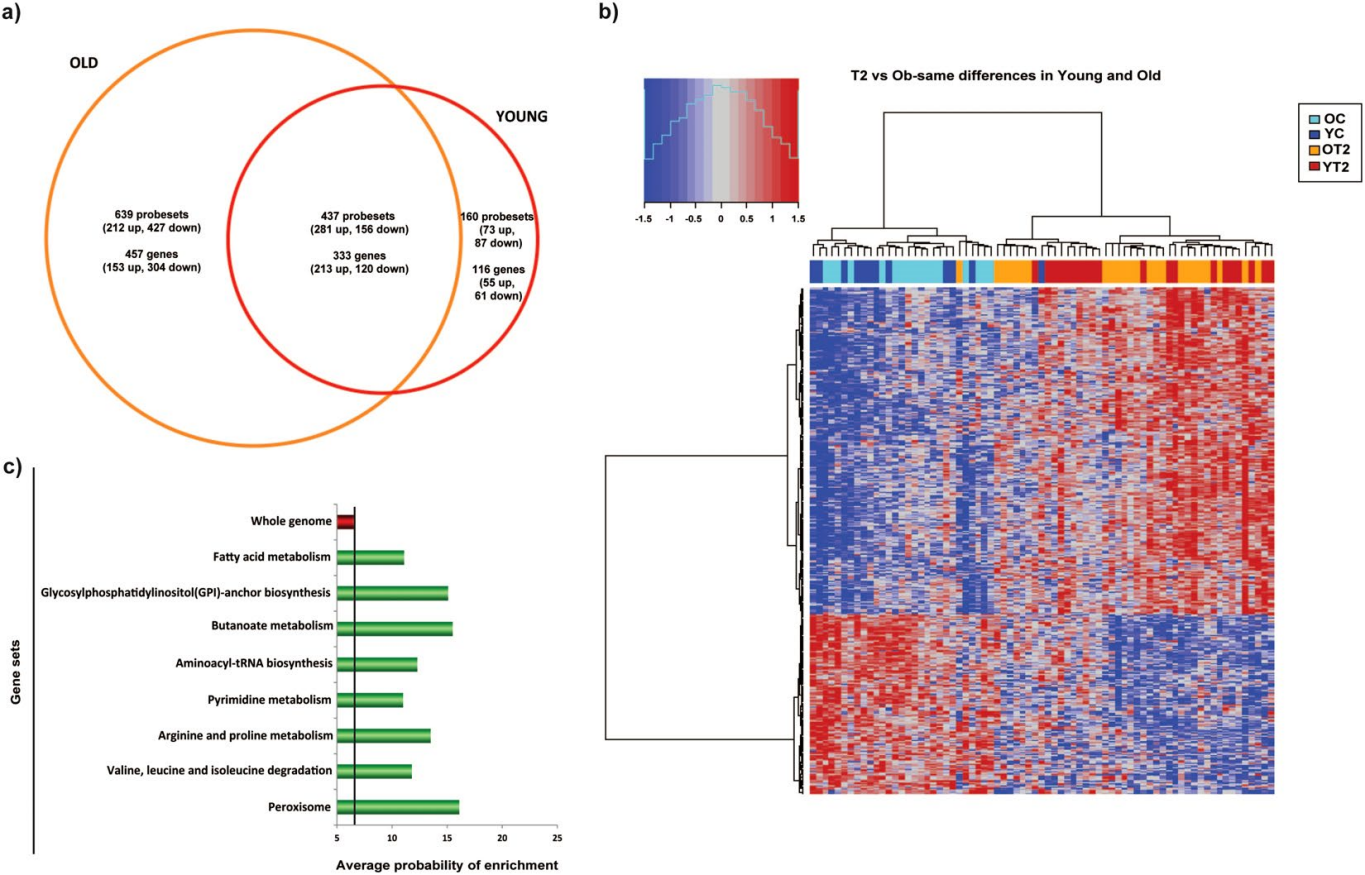
Early-onset and classical forms of type 2 diabetes generate a gene signature. (**a**) Venn diagram depicting genes differentially expressed in muscle from type 2 diabetic patients compared with muscle from age- and BMI-matched nondiabetic subjects. (**b**) Heatmap of genes differentially expressed in muscle from type 2 diabetic patients. YT2 are early-onset type 2 diabetic subjects; OT2 are older type 2 diabetic subjects, and their respective controls are YC and OC. (**c**) GSEA of genes dysregulated in muscles from type 2 diabetic patients.

The global expression profile of muscles from control (YC and OC) and type 2 diabetic patients (YT2, and OT2) was identified by microarray analysis. In classical type 2 diabetic patients we identified 790 genes that underwent dysregulation (366 over-expressed, and 424 under-expressed) (Supplementary Table 1), whereas in early-onset type 2 diabetic patients we identified 449 dysregulated genes (268 over-expressed, and 181 under-expressed) (Supplementary Table 1). Interestingly, a significant proportion of genes (333) were dysregulated in both groups of patients (pv 2.22e-16). From those, 213 genes showed over-expression in type 2 diabetic patients while 120 were under-expressed compared to control subjects (Fig. 1a and Supplementary Table 1). As a result of this analysis, a clear gene signature in skeletal muscle from type 2 diabetes patients is seen in the clustering heatmap shown in Fig. 1b. We acknowledge some limitations in our study namely sex unbalance between obese and type 2 diabetic subjects. These limitations have been partially neutralized by adjusting microarray data for gender. Gene Set Enrichment Analysis (GSEA) revealed that genes dysregulated in both classical and early-onset type 2 diabetic patients were enriched in metabolic pathways such as the metabolism of amino acids (BCAA, arginine and proline), fatty acids, pyrimidines or peroxisomes (Fig. 1c).

### Weighted Co-expression Networks Analysis (WGCNA) reveals the relevance of the BCAA genes in the pathophysiology of type 2 diabetes

**Figure 2:**
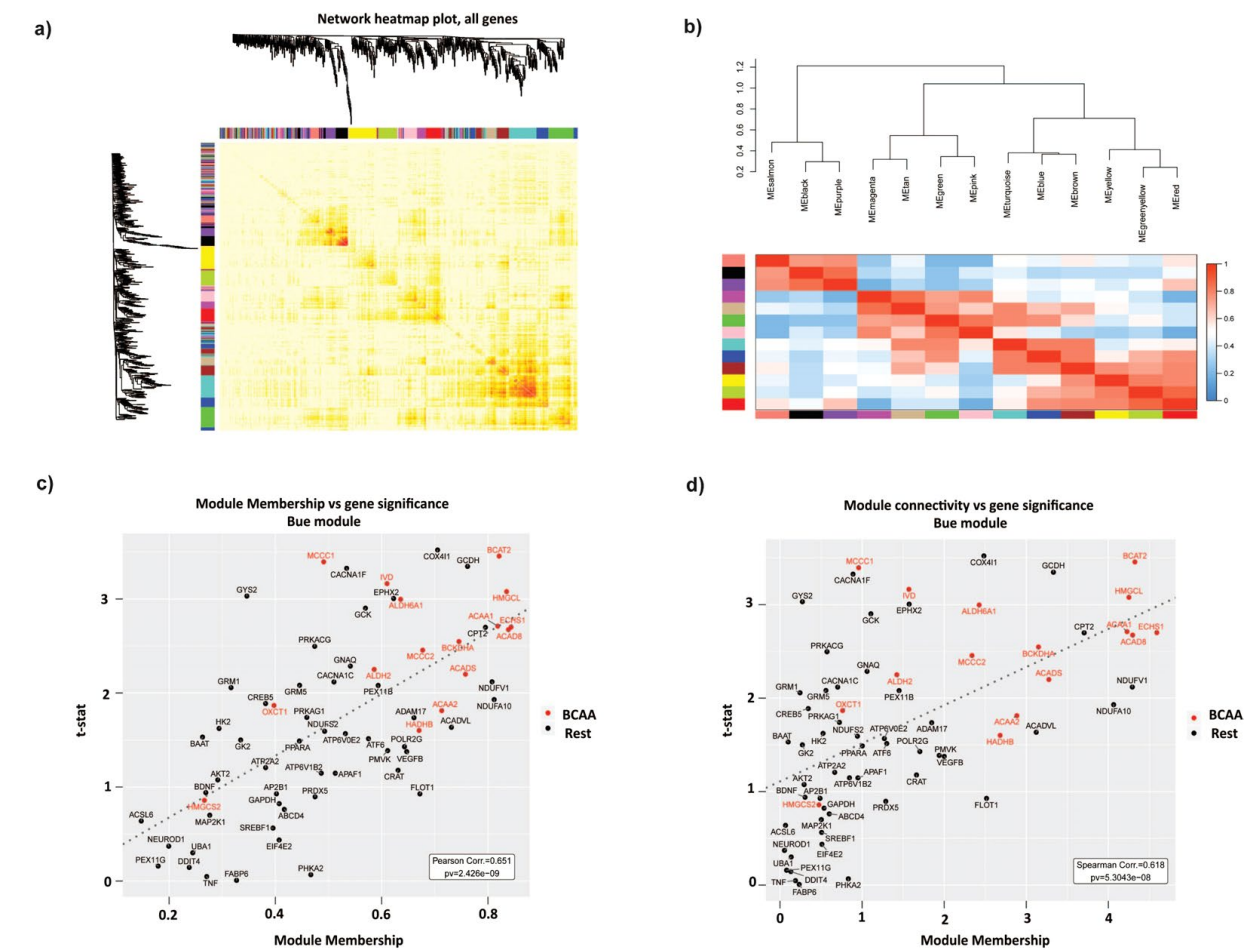
Identification of gene co-expression modules in human skeletal muscle from control and type 2 diabetic patients by WGCNA analysis. (**a**) Network heatmap plot. Branches of the cluster dendrogram of the most connected genes gave rise to a number of gene co-expression modules. (**b**) Heatmap plot of the adjacencies in the eigengene network. Each row and column in the heatmap corresponds to one module eigengene (labeled by color). In the heatmap, blue color represents low adjacency (negative correlation), while red represents high adjacency (positive correlation). Panels c, and d. Scatterplots of gene significance versus module connectivity (panel c) or module membership (panel d) in the Blue module. BCAA genes are shown in red color.

To analyze the gene signature found for type 2 diabetes patients in a deep manner we decided to mix control groups or type 2 diabetic groups independently of age (young and old together). The clinical and metabolic characteristics of this rearrangement are presented in Table 2. Next, we analyzed the potential relevance of BCAA genes and the interaction between BCAA metabolic genes and other metabolic genes by Weighted Co-expression Networks Analysis (WGCNA). WGCNA is a useful tool to describe the pairwise relationships among gene transcripts and to unravel genetic mechanisms of complex diseases^30–32^. Thus, we selected the most relevant gene sets obtained in GSEA (Supplementary Table 2), and the genes of those categories were subjected to WGCNA. This permitted the generation of a gene co-expression network (Fig. 2a). The TOM heatmap graph shows the existence of co-expression interconnectedness, which is indicated by progressively more saturated yellow and red colors, and a total of 13 modules were identified (different colors in Fig. 2a). Clustering Analysis on Eigengene Network adjacencies revealed the existence of module relationships and defined four different groups of modules (meta-modules) (Fig. 2b): Red, Greenyellow and Yellow modules (meta-module A); Brown, Blue and Turquoise (meta-module B); Pink, Green, Etan and Magenta (meta-module C); and Purple, Black and Salmon modules (meta-module D). Most of the modules in meta-modules A and B showed a significant association with type 2 diabetes and a strong correlation between gene module-membership (MM), connectivity and gene significance, the later measured as the t-statistic from a linear model (Fig. 2c,d for the Blue module). From those, the Blue module was enriched in gene sets of BCAA and Fatty acid metabolism genes (Supplementary Table 3). Interestingly, the Green module was also enriched in BCAA genes although no association with type 2 diabetes was found. A graph representation with the strongest connections of the blue module genes highlight the central role of BCAA genes as BCAT2, HMGCL, ACAA2, ACAD8, HADHB or ACADS in this sub-network (Supplementary Figure 1). In all, this statistical analysis further suggests the relevance of the BCAA genes in the pathophysiology of type 2 diabetes.

**Table 2:** Anthropometric and metabolic parameters measured in control and Type 2 diabetic subjects.

	Control subjects	Type 2 diabetes subjects
n	29	45
Male/female ratio	16/13	32/13
Age (years)	42 ± 16.45	42.6 ± 14.49
BMI (kg/m2)	34.3 ± 4.15	35.09 ± 7.36
Fasting glucose (mmol/l)	5.02 ± 0.60	9.36 ± 2.74*
Fasting insulin (pmol/l)	19.46 ± 30.2	29.45 ± 28.5
VO2max (ml*kg-1*min-1)	21.88 ± 5.02	22.97 ± 5.25

Data are means ± standard deviation. Statistical analyses comparing control and type 2 diabetes state were performed by unpaired *t*-Test.

### Type 2 diabetes is characterized by reduced muscle expression genes encoding enzymes of BCAA metabolism

**Figure 3:**
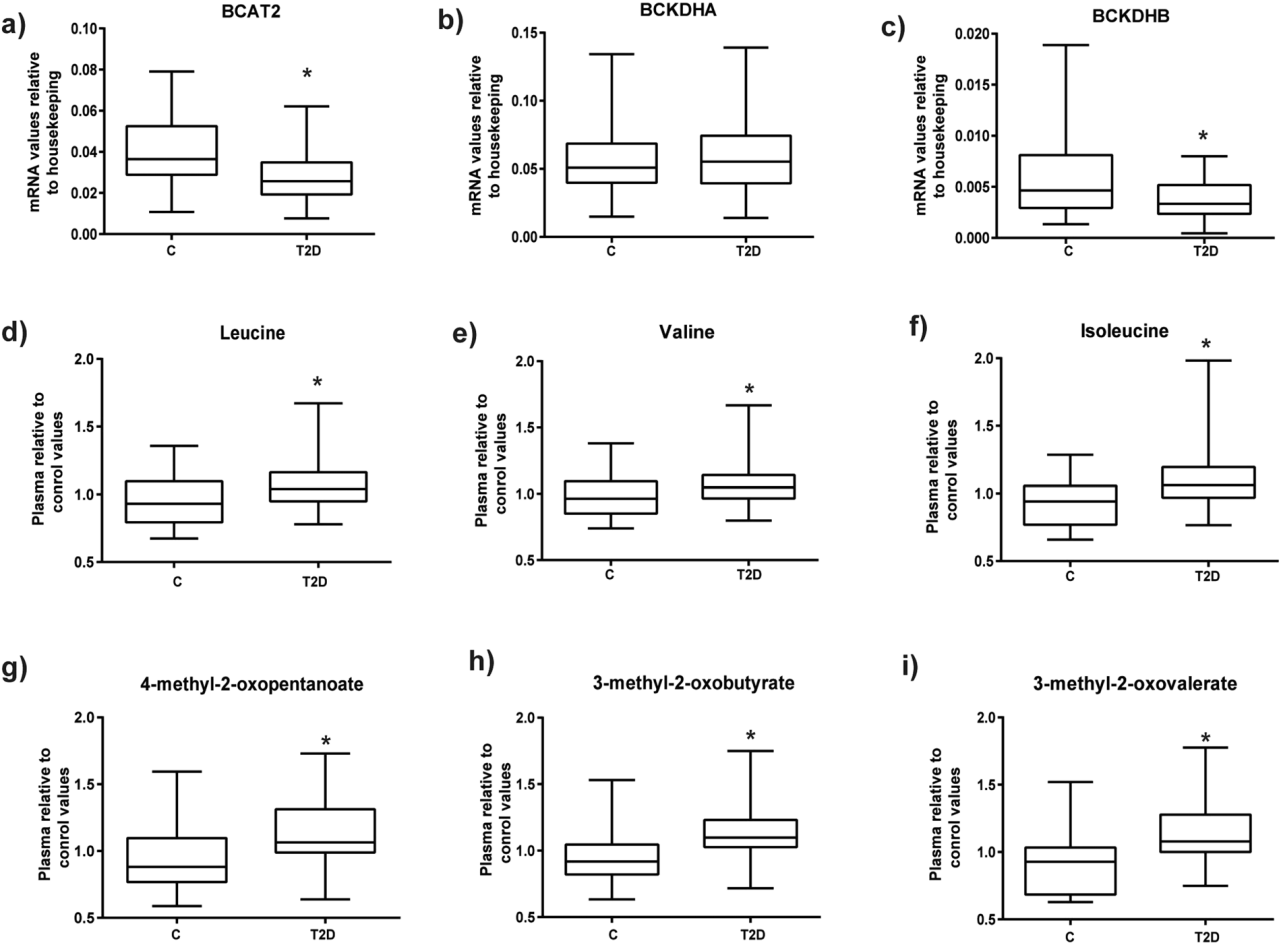
Skeletal muscle gene expression of branched chain amino acids mitochondrial proteins in type 2 diabetic subjects (T2D) and their respective matched control groups (C). Real-time PCR was performed in skeletal muscle biopsies: **(a**) BCAT2, (**b**) BCKDHA, and (**c**) BCKDHB); Plasma metabolomics was performed in the same subjects and relative values are shown: (**d**) Leucine, (**e**) Valine, (**f**) Isoleucine and catabolites (**g**) 4-methyl-2-oxopentanoate, (**h**) 3-methyl-2-oxobutyrate and (**i**) 3-methyl-2-oxovalerate. Data are presented as mean ± standard deviation. Statistical analyses comparing type 2 diabetic subjects vs respective controls were performed by unpaired *t*-test *p < 0.05.

Based on the GSEA and WGCNA data obtained in previous sections, we next performed real-time PCR assays to analyze the expression of genes encoding enzymes of BCAA metabolism in muscle from the pooled control and type 2 diabetic groups to validate the microarray analysis. As shown in Fig. 3, when compared to BMI-matched obese controls, type 2 diabetes is characterized by reduced expression of genes encoding for the mitochondrial steps of BCAA metabolism BCAT2, and BCKDHB (encoding the E1beta subunit of BCKD) (Fig. 3a,b). However, no changes were observed in the mRNA expression of BCKDHA in the diabetic groups (Fig. 3b). To further analyze the nature of the defective BCAA gene expression in diabetic muscle, the same group of patients was analyzed for differential methylation in genes involved in BCAA metabolism. This analysis revealed an increased methylation in the gene encoding the E1beta subunit of BCKD in type 2 diabetic patients (Supplementary Figure 2), which is consistent with the reduced gene expression detected under these conditions (Fig. 3c).

We also analyzed the groups separately according to age, as shown in Supplementary Figure 3. Early-onset type 2 diabetes was characterized by reduced expression of BCAT2 (Supplementary Figure 3 B, C). In type 2 diabetes of older subjects a reduced expression was also detected for BCKDHB (Supplementary Figure 3 C). Thus, both classical and early-onset type 2 diabetes are characterized by reduced skeletal muscle expression of BCAA genes.

### Plasma metabolomics supports the view for a defect in branched-chain amino acid handling in type 2 diabetes

Metabolomic analysis performed in plasma of the same subjects, showed a significant accumulation of BCAAs in type 2 diabetes compared with non-diabetic subjects. Plasma leucine, valine, and isoleucine levels were elevated in subjects with type 2 diabetes relative to control group (Fig. 3d–f). More importantly, the 2-keto acids of all BCAAs (BCKAs) (3-methy-2-oxobutyrate, 3-methyl-2-oxovalerate, and 4-methyl-2-oxopentanoate), which are the substrates for BCKD, were also markedly elevated in plasma from type 2 diabetics (Fig. 3g–i).

When we analyzed the groups according to age, no significant differences for BCAAs and their 2-keto acids was observed for early onset type 2 diabetes and old subjects with type 2 diabetes. Only 3-methyl-2-oxobutyrate levels were increased statistically significant in both groups of diabetic subjects (Supplementary Figure 3 D –I). Old type 2 diabetic subjects also showed statistically greater levels of valine, isoleucine, and the other 2-keto acids (4-methyl-2-oxopentanoate and 3-methyl-2oxovalerate) (Supplementary Figure 3 D –I).

### Plasma BCAAs and their catabolites negatively correlate with gene expression of BCAA enzymes in type 2 diabetic patients

**Figure 4:**
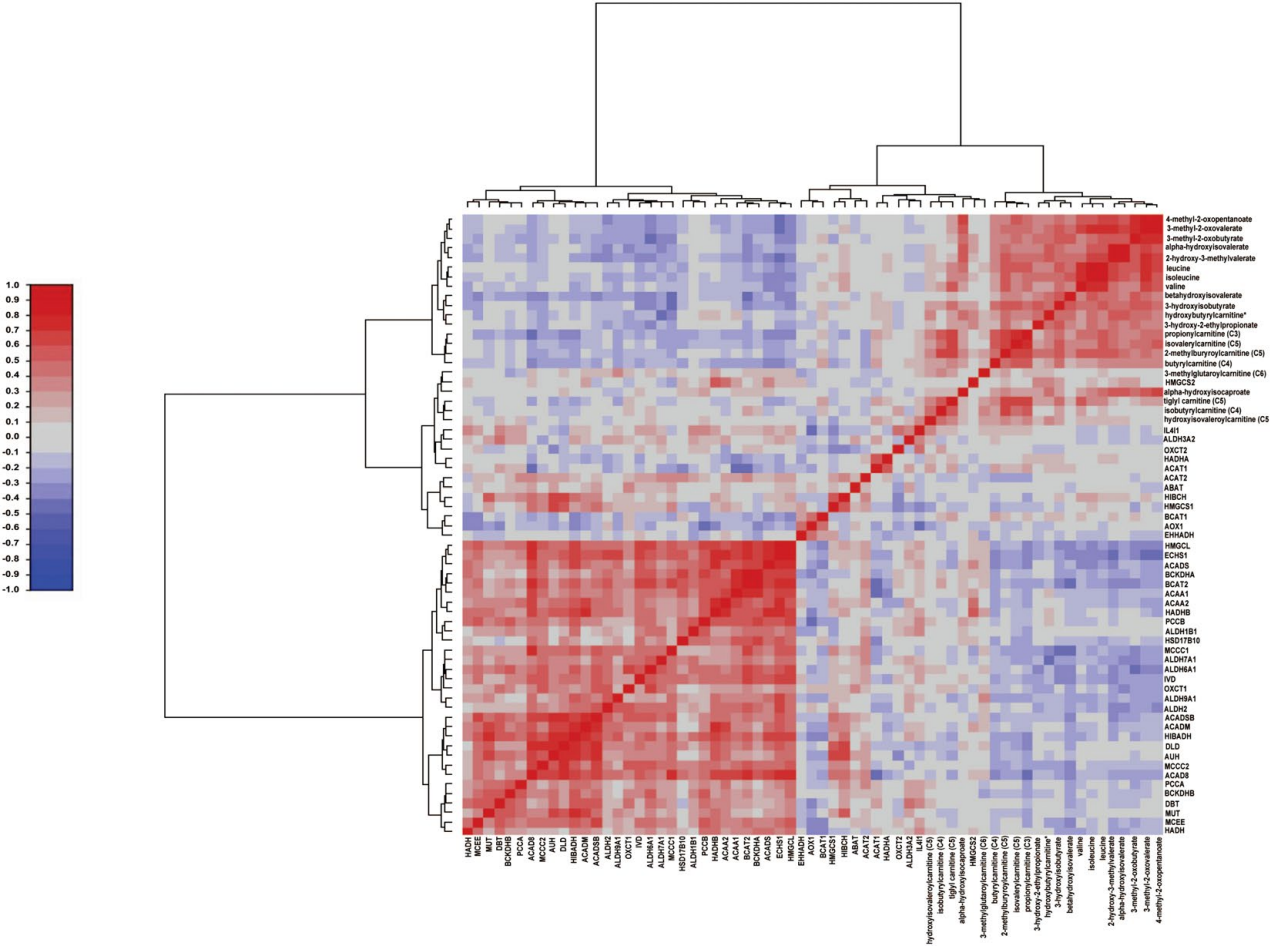
Plasma metabolomics correlation with skeletal muscle gene expression. Correlation analysis of plasma branched chain amino acids and their catabolites with gene expression (obtained in microarray) of enzymes involved in the catabolism of branched chain amino acids in skeletal muscle. Statistics is shown in Supplementary Table 5.

To get some insight into the capacity of the T2D patients to metabolize BCAA as compared to non-diabetic subjects, correlation analyses were performed between plasma BCAA and their corresponding catabolites detected by metabolomics, and the microarray data from muscle transcriptomics. Results for these analyses showed a negative correlation between most of the plasma metabolites and transcripts linked to BCAA metabolism in skeletal muscle (Fig. 4, and Supplementary Table 4). In addition, similar correlations were detected when plasma metabolomics were analyzed with real time PCR expression levels of transcripts encoding for BCAA catabolizing enzymes assessed in skeletal muscle biopsies. (Supplementary Figure 4, and Supplementary Table 5). These data permit to propose that alterations in the expression of genes encoding enzymes involved in BCAA metabolism lead to enhanced release and greater circulating levels of BCAA and their metabolites.

### Ob/ob obese mice show elevated plasma BCAAs and alter protein levels of BCAA enzymes in liver and adipose tissue but not in muscle

**Figure 5:**
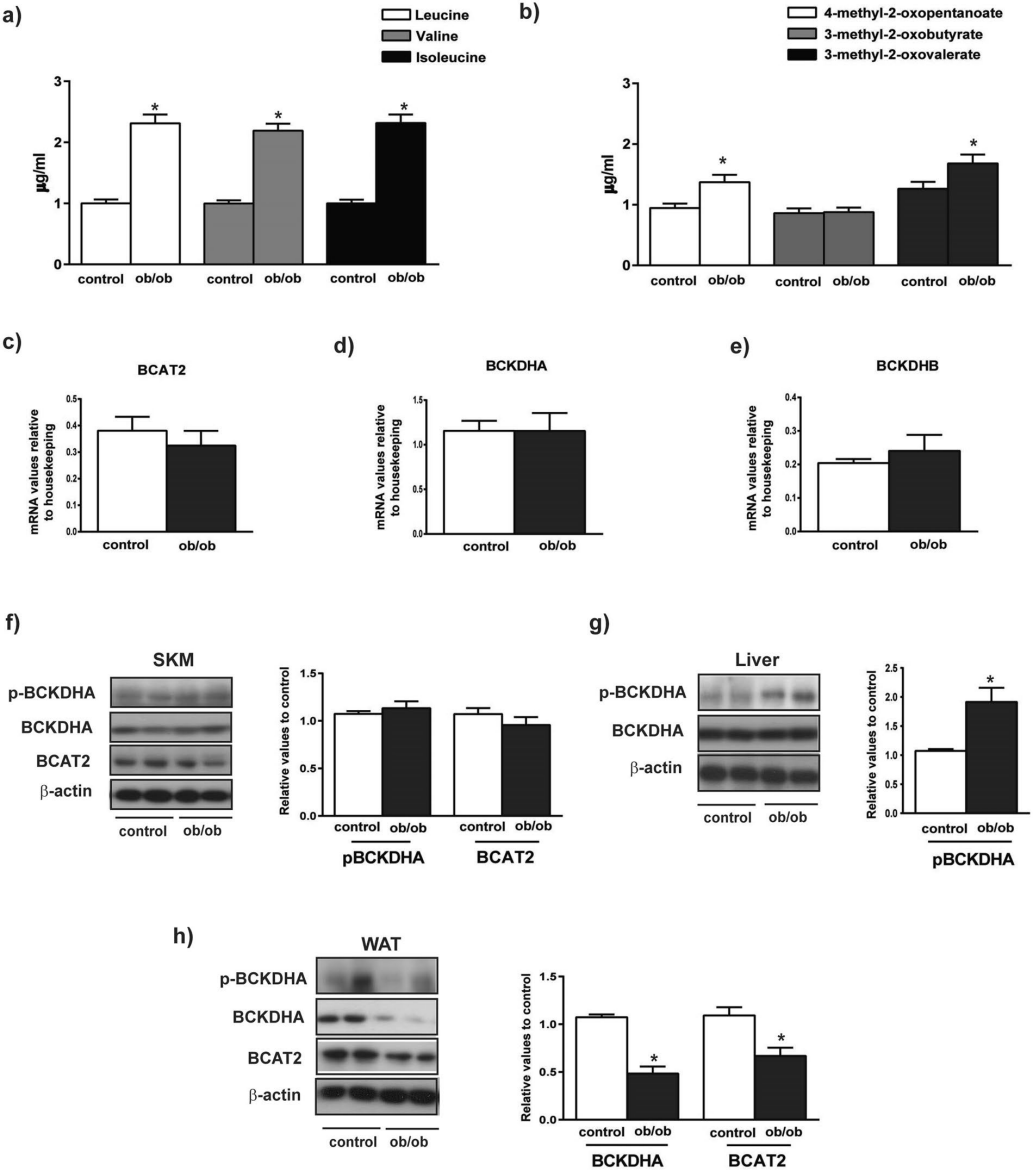
Characterization of obese, insulin resistant ob/ob mice. (**a**) Plasma content of branched chain amino acids from control and ob/ob mice. (**b**) Plasma content of keto acids from control and ob/ob mice. (**c**–**e**) mRNA relative values of mitochondrial genes involved in catabolism of branched chain amino acids in skeletal muscle from control and ob/ob mice. (**f**) Skeletal muscle (SKM) phosphorylation, protein levels and quantification of mitochondrial branched chain amino acids catabolic enzymes from control and ob/ob mice. (**g**) Liver phosphorylation, protein levels and quantification of mitochondrial branched chain amino acids catabolic enzymes from control and ob/ob mice. (**h**) White adipose tissue (WAT) phosphorylation, protein levels and quantification of mitochondrial branched chain amino acids catabolic enzymes from control and ob/ob mice. The images are cropped from full blots, which are shown in Supplementary Information. Data are means ± SEM. Statistical analyses comparing ob/ob mice vs controls were performed by unpaired *t*-test *p < 0.05.

To document whether the situation on BCAAs in human subjects was similar to that occurring in mouse models of obesity/insulin resistance, we analyzed the profile of BCAAs in obese ob/ob mice and their respective lean controls. To do so we performed focused metabolomics in plasma and measured the expression of BCAA metabolizing enzymes in skeletal muscle, liver and white adipose tissue (WAT). As shown in Fig. 5a, the plasma levels of BCAA leucine, valine and isoleucine were two-fold higher in ob/ob mice compared to their controls. Moreover, increased plasma levels of 4-methyl-2 oxopentanoate (BCKA derived from leucine) and 3-methyl-2 oxovalerate (BCKA from isoleucine) were also detected in ob/ob mice (Fig. 5b). However no changes were found in 3-methyl-2-oxobutyrate which is the BCKA derived from valine (Fig. 5b).

Expression of the genes encoding BCAA degradation enzymes such as the mitochondrial transaminase (BCAT2) and the subunits alpha and beta of BCKD (BCKDHA and BCKDHB) was unaltered in muscle from ob/ob mice compared with controls (Fig. 5c–e). To analyze the potential impact of methylation on gene expression, we performed quantitative analysis of methylation in genes encoding for BCAA metabolism. Data revealed that the BCAT2 gene is highly methylated in the livers of control mice (Supplementary Figure 5 A,C), which is consistent with the lack of BCAT2 expression in liver. No changes in methylation of BCAT2 gene was detected in livers from ob/ob mice (Supplementary Figure 5E). The BCAT2 gene was not methylated in muscle or in white adipose tissue, which was in parallel to a substantial gene expression (Supplementary Figure 5D). BCKDHA gene was barely methylated in all tissues studied (Supplementary Figure 5B).

According with prior reports^33^, no changes in the muscle of BCAT2 or BCKDHA proteins levels or in the phosphorylation of the subunit E1a of BCKDHA complex were detected in ob/ob respect to control mice (Fig. 5f). However, the phosphorylation level of BCKDHA was twofold greater in the liver from ob/ob mice, which indicates a lower activity compared to controls (Fig. 5g). In WAT we detected a reduced expression of BCKDHA and BCAT2 in ob/ob mice (Fig. 5h).

Overall, our results support the view that the higher plasma levels of BCAAs detected in obesity/insulin resistance ob/ob mice are a consequence of reduced protein expression of BCAA metabolism enzymes in liver and white adipose tissue, and occur in the absence of alterations in skeletal muscle.

### Db/db diabetic mice show alterations in muscle protein levels of BCAA degradation enzymes and elevated plasma BCAA *α-keto* acids

**Figure 6:**
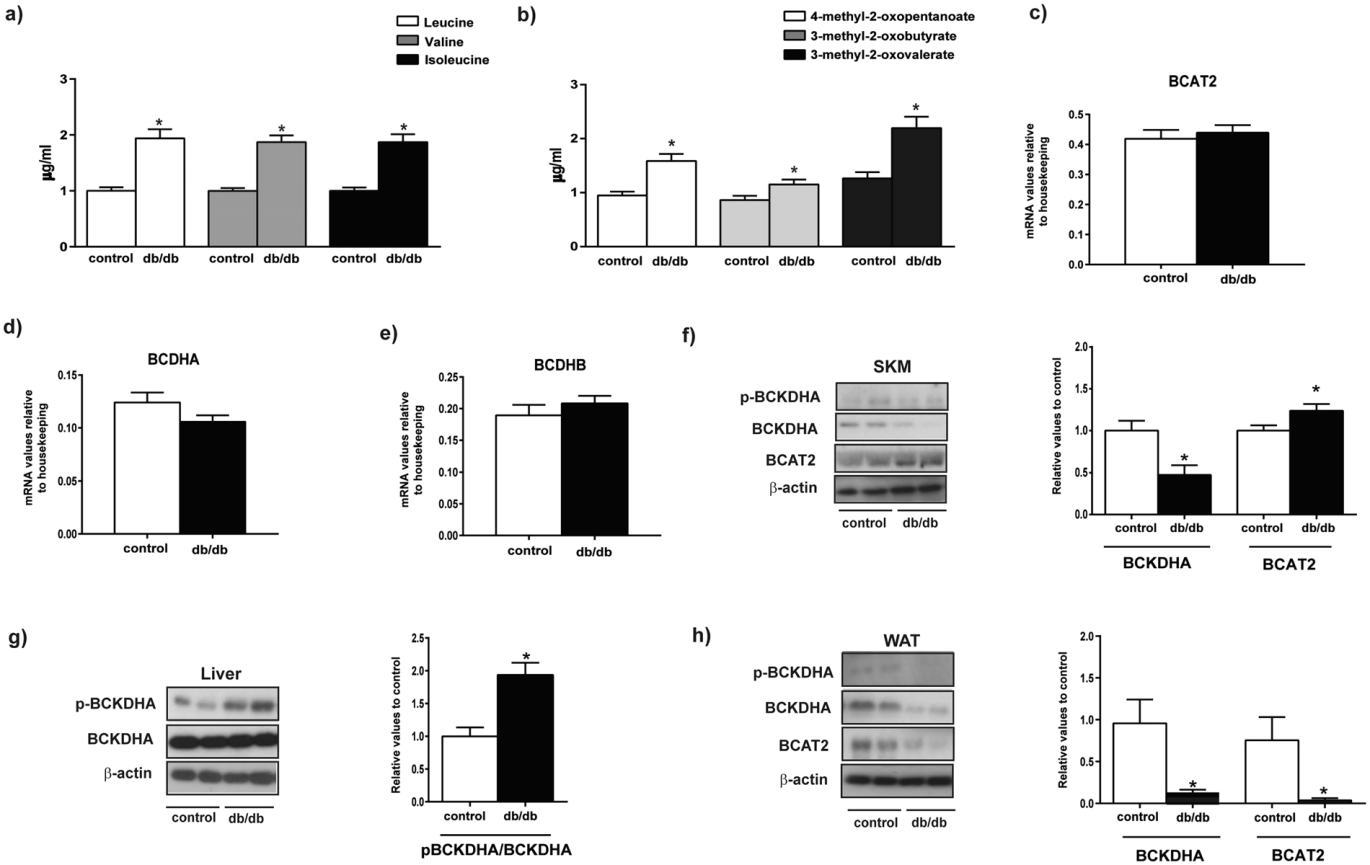
Characterization of diabetic db/db mice. (**a**) Plasma content of branched chain amino acids from control and db/db mice. (**b**) Plasma content of keto acids from control and db/db mice. (**c**–**e**) mRNA relative values of mitochondrial genes involved in catabolism of branched chain aminoacids in skeletal muscle from control and db/db mice. (**f**) Skeletal muscle (SKM) phosphorylation, protein levels and quantification of mitochondrial branched chain aminoacids catabolic enzymes from control and db/db mice. (**g**) Liver phosphorylation, protein levels and quantification of mitochondrial branched chain aminoacids catabolic enzymes from control and db/db mice. (**h**) White adipose tissue (WAT) phosphorylation, protein levels and quantification of mitochondrial branched chain amino acids catabolic enzymes from control and db/db mice. The images are cropped from full blots, which are shown in Supplementary Information. Data are mean ± SEM. Statistical analyses comparing control vs db/db mice were performed by unpaired *t*-test *p < 0.05.

Next, we performed focused plasma metabolomics and assayed the expression of BCAA metabolizing enzymes in diabetic db/db mice compared to control mice. Plasma levels of BCAAs, leucine and isoleucine were enhanced in db/db mice compared to the control group (Fig. 6a). In addition, plasma levels of all of three alpha-keto acids (4-methyl-2 oxopentanoate, 3-methyl-2-oxobutyrate and 3-methyl-2 oxovalerate) were increased in db/db mice (Fig. 6b). Further statistical analysis revealed a slight decrease in the plasma levels of isoleucine in db/db animals compared to ob/ob, that was in parallel to the increase observed in the 3-methyl-2-oxovalerate (the alpha-ketoacid of isoleucine) (Supplementary Figure 6). The pattern of changes in BCAA and BCKA found in diabetic mice was very similar to the signature detected in human type 2 diabetes.

In keeping with the observations in ob/ob mice, the phosphorylation level of BCKDHA was twofold greater in livers from db/db mice, which indicates a lower activity (Fig. 6g), and a reduced expression of BCKDHA and BCAT2 in WAT were detected in db/db mice (Fig. 6h).

In skeletal muscle, the expression of genes encoding BCKDHA and BCKDHB only showed a trend to decrease in the db/db group (Fig. 6c–e), and the total levels of BCKDHA was reduced in db/db mice (Fig. 6f) (50% reduced compared to the control group). Under these conditions, an increased muscle BCAT2 protein was detected in db/db mice (Fig. 6f). Our data indicate that diabetic db/db mice may be a good model of type 2 diabetes, and are characterized by reduced muscle BCAA degradation due to a lower level of BCKDHA subunit complex, and increased levels of the transaminase (BCAT2). This may explain the greater plasma levels of BCKAs found in db/db mice and in type 2 diabetic patients.

## Discussion

Early-onset type 2 diabetes is typically characterized by obesity and severe insulin resistance in young people with a strong family history of type 2 diabetes^34,35^. In prior publications, we reported that early-onset type 2 diabetic patients do not improve VO_2_max in response to an exercise intervention, and in parallel, acute exercise does not induce muscle PGC-1alpha gene expression, and chronic exercise does not induce muscle mitochondrial Mfn2 expression^12,13^. However, almost nothing is known about the physiology of the skeletal muscle on early-onset type 2 diabetes.

The metabolism of BCAAs is a highly regulated process, and loss of ability to metabolize them causes maple syrup urine disease in humans^36^. Skeletal muscle is also the major organ extracting an intravenous leucine^37^. Muscle has the capacity to oxidize BCAAs and BCKAs or to release their metabolites into the circulation. Thus, it has been recently identified that 3-hydroxyisobutyrate is released from muscle and causes insulin resistance^27^. Our results revealed increased circulating concentrations of BCAA and their alpha-keto acids as well as a reduced expression of genes involved in BCAA metabolism. In addition, our data showed a negative correlation between the increased levels of plasmatic BCAAs, and their metabolites, and the reduced expression of genes encoding BCAA enzymes in type 2 diabetes. This suggests that the alterations occurring in muscle have an impact on the circulating levels of BCAAs and their metabolites. Then, we propose that different forms of type 2 diabetes or progression to different forms of type 2 diabetes reduce the handling of BCAAs in skeletal muscle. An impaired mitochondrial BCAA catabolism in skeletal muscle could also cause a potential release of BCAAs and their BCKAs from muscle to the circulation in type 2 diabetic conditions.

Our data in mice models support the view that skeletal muscle does not alter BCAA gene expression under conditions of insulin resistance alone. In this respect, it operates differentially from liver and adipose tissues which reduce BCAAs metabolism under insulin resistant conditions. Instead, BCAA metabolism is reduced in skeletal muscle during the stage of frank type 2 diabetes. In this connection, it has been reported that branched chain keto acids (BCKAs) can inactivate 2-ketoglutarate and pyruvate dehydrogenase complexes in muscle^38^. In addition, the effects of BCKAs inhibits pyruvate dehydrogenase flux leaving the most of the pyruvate dehydrogenase in the inactive form in liver^39^ and since the mitochondrial pyruvate dehydrogenase complex (PDC) controls the rate of carbohydrate oxidation, impairment of PDC activity mediated by high-fat intake has been advocated as a causative factor for the skeletal muscle insulin resistance, metabolic syndrome, and the onset of type 2 diabetes (T2D)^40^.

In contrast, studies performed in C. elegans indicate that silencing of the gene ortologous to BCAT1 enhances lifespan, which is dependent on higher levels of BCAAs, and neuroendocrine repression of TGFbeta (Mansfeld *et al*., Nat. Commun. 2015). This suggests the possibility that enhanced levels of BCAAs and their alpha-keto acids may show benefitial effects in humans.

In agreement with our results, Giesbertz and colleagues found that plasmatic levels of BCAA were upregulated in both ob/ob and db/db mice^41^. Regarding BCAA metabolites, Giesbertz *et al*. documented enhanced plasma levels of 3-hydroxyisobutyrate (a metabolite of the BCAA degradation pathway) in db/db mice but not in the ob/ob group. In addition, they found enhanced plasma levels of α-ketoisocaproic acid (the alpha-ketoacid from leucine) both in ob/ob and in db/db mice, while our results only showed a moderate increase in db/db mice compared to control. The differences between Giesbertz *et al*. and our study could be explained by the age of the mice. Thus, Giesbertz and colleagues studied mice at 20 weeks of age, whereas our analysis was done at 8 weeks of age, and it is likely that insulin resistance gets worse with age leading to larger differences in plasma metabolites. Another interesting angle covered by Giesbert *et al*., was the correlation analysis between plasma and tissue metabolites in ob/ob and db/db^41^. This analysis revealed a correlation between BCAAs and metabolites in plasma and adipose tissue or in liver suggesting a role of these tissues in the alterations detected in BCAA metabolites in plasma. Our data also support the view that the higher plasma levels of BCAAs detected in obesity/insulin resistance ob/ob mice are a consequence of reduced protein expression of BCAA metabolism enzymes in liver and white adipose tissue. In addition, our data indicate that diabetic db/db mice are characterized by reduced muscle BCAA degradation due to a lower level of BCKDHA subunit complex, and increased levels of the transaminase (BCAT2), and this may explain the greater plasma levels of BCKAs found in db/db mice.

In summary, our data indicate that classical or early-onset forms of type 2 diabetes are characterized by reduced expression of genes involved in BCAA metabolism. In addition, type 2 diabetes is linked to inhibition of BCKD complex in skeletal muscle. These alterations are also detected in muscles from diabetic db/db mice but not in obese ob/ob mice. Years ago the pyruvate dehydrogenase complex (PDC) was reported as an emerging target for the treatment of metabolic syndrome^42^. However, now we know that BCKAs inhibits its activity at least in muscle and liver^38,39^. In this regard, our data suggest that the alterations in muscle BCAA handling develop during the progression of type 2 diabetes, are relevant to cause the higher concentrations in circulating BCKAs.

## Electronic supplementary material

Supplementary data

## References

[1] DeFronzo RA, Tripathy D (2009). Skeletal Muscle Insulin Resistance Is the Primary Defect in Type 2 Diabetes. Diabetes Care.

[2] Turcotte LP, Fisher JS (2008). Skeletal Muscle Insulin Resistance: Roles of Fatty Acid Metabolism and Exercise. Physical Therapy.

[3] Fröjdö S, Vidal H, Pirola L (2009). Alterations of insulin signaling in type 2diabetes: A review of the current evidence from humans. Biochimica et Biophysica Acta (BBA) - Molecular Basis of Disease.

[4] Krook A, Wallberg-Henriksson H, Zierath JR (2004). Sending the signal: molecular mechanisms regulating glucose uptake. Med Sci Sports Exerc.

[5] Abdul-Ghani, M. A. & DeFronzo, R. A. Pathogenesis of Insulin Resistance in Skeletal Muscle. *Journal of Biomedicine and Biotechnology* **2010**, doi:10.1155/2010/476279 (2010). 10.1155/2010/476279 PMC2860140 20445742

[6] Shulman GI (1990). Quantitation of Muscle Glycogen Synthesis in Normal Subjects and Subjects with Non-Insulin-Dependent Diabetes by 13C Nuclear Magnetic Resonance Spectroscopy. New England Journal of Medicine.

[7] Schrauwen-Hinderling VB, Roden M, Kooi ME, Hesselink MK, Schrauwen P (2007). Muscular mitochondrial dysfunction and type 2 diabetes mellitus. Current opinion in clinical nutrition and metabolic care.

[8] Kelley DE, He J, Menshikova EV, Ritov VB (2002). Dysfunction of mitochondria in human skeletal muscle in type 2 diabetes. Diabetes.

[9] Patti ME (2003). Coordinated reduction of genes of oxidative metabolism in humans with insulin resistance and diabetes: Potential role of PGC1 and NRF1. Proceedings of the National Academy of Sciences of the United States of America.

[10] Mootha VK (2003). PGC-1alpha-responsive genes involved in oxidative phosphorylation are coordinately downregulated in human diabetes. Nature genetics.

[11] Hagiwara N (2014). Genetic Dissection of the Physiological Role of Skeletal Muscle in Metabolic Syndrome. New Journal of Science.

[12] Burns N (2007). Early-onset type 2 diabetes in obese white subjects is characterised by a marked defect in beta cell insulin secretion, severe insulin resistance and a lack of response to aerobic exercise training. Diabetologia.

[13] Hernandez-Alvarez MI (2010). Subjects with early-onset type 2 diabetes show defective activation of the skeletal muscle PGC-1{alpha}/Mitofusin-2 regulatory pathway in response to physical activity. Diabetes Care.

[14] Lu J, Xie G, Jia W (2013). Insulin resistance and the metabolism of branched-chain amino acids. Front Med.

[15] Huffman KM (2009). Relationships between circulating metabolic intermediates and insulin action in overweight to obese, inactive men and women. Diabetes Care.

[16] Tai ES (2010). Insulin resistance is associated with a metabolic profile of altered protein metabolism in Chinese and Asian-Indian men. Diabetologia.

[17] Newgard CB (2009). A branched-chain amino acid-related metabolic signature that differentiates obese and lean humans and contributes to insulin resistance. Cell metabolism.

[18] Fiehn O (2010). Plasma metabolomic profiles reflective of glucose homeostasis in non-diabetic and type 2 diabetic obese African-American women. PloS one.

[19] Wang TJ (2011). Metabolite profiles and the risk of developing diabetes. Nature medicine.

[20] Adams SH (2011). Emerging Perspectives on Essential Amino Acid Metabolism in Obesity and the Insulin-Resistant State. Advances in Nutrition.

[21] Newgard CB (2012). Interplay between lipids and branched-chain amino acids in development of insulin resistance. Cell metabolism.

[22] Yoon M-S, Choi CS (2016). The role of amino acid-induced mammalian target of rapamycin complex 1(mTORC1) signaling in insulin resistance. Exp Mol Med.

[23] Shimobayashi M, Hall MN (2016). Multiple amino acid sensing inputs to mTORC1. Cell Res.

[24] Shimizu N (2011). Crosstalk between glucocorticoid receptor and nutritional sensor mTOR in skeletal muscle. Cell metabolism.

[25] Laplante M, Sabatini DM (2012). mTOR signaling in growth control and disease. Cell.

[26] Zhang Y (2014). Coordinated regulation of protein synthesis and degradation by mTORC1. Nature.

[27] Jang, C. *et al*. A branched-chain amino acid metabolite drives vascular fatty acid transport and causes insulin resistance. *Nature medicine*, doi:10.1038/nm.4057 (2016). 10.1038/nm.4057 PMC4949205 26950361

[28] Lerin C (2016). Defects in muscle branched-chain amino acid oxidation contribute to impaired lipid metabolism. Molecular metabolism.

[29] Irizarry RA (2003). Summaries of Affymetrix GeneChip probe level data. Nucleic acids research.

[30] Langfelder P, Horvath S (2007). Eigengene networks for studying the relationships between co-expression modules. BMC systems biology.

[31] Langfelder P, Horvath S (2008). WGCNA: an R package for weighted correlation network analysis. BMC bioinformatics.

[32] Horvath S (2006). Analysis of oncogenic signaling networks in glioblastoma identifies ASPM as a molecular target. Proceedings of the National Academy of Sciences of the United States of America.

[33] She P (2007). Obesity-related elevations in plasma leucine are associated with alterations in enzymes involved in branched-chain amino acid metabolism. Am J Physiol Endocrinol Metab.

[34] Fagot-Campagna A (2000). Type 2 diabetes among North American children and adolescents: an epidemiologic review and a public health perspective. The Journal of pediatrics.

[35] McQuaid S (2005). Early-onset insulin-resistant diabetes in obese Caucasians has features of typical type 2 diabetes, but 3 decades earlier. Diabetes Care.

[36] Burrage LC, Nagamani SC, Campeau PM, Lee BH (2014). Branched-chain amino acid metabolism: from rare Mendelian diseases to more common disorders. Human molecular genetics.

[37] Hagenfeldt L, Eriksson S, Wahren J (1980). Influence of leucine on arterial concentrations and regional exchange of amino acids in healthy subjects. Clin Sci (Lond).

[38] Jackson RH, Singer TP (1983). Inactivation of the 2-ketoglutarate and pyruvate dehydrogenase complexes of beef heart by branched chain keto acids. J Biol Chem.

[39] Walajtys-Rode E, Williamson JR (1980). Effects of branched chain alpha-ketoacids on the metabolism of isolated rat liver cells. III. Interactions with pyruvate dehydrogenase. J Biol Chem.

[40] Constantin-Teodosiu D (2013). Regulation of muscle pyruvate dehydrogenase complex in insulin resistance: effects of exercise and dichloroacetate. Diabetes Metab J.

[41] Giesbertz P (2015). Metabolite profiling in plasma and tissues of ob/ob and db/db mice identifies novel markers of obesity and type 2 diabetes. Diabetologia.

[42] Lee I-K (2014). The Role of Pyruvate Dehydrogenase Kinase in Diabetes and Obesity. Diabetes & Metabolism Journal.

