# Supplementary material for: Early-onset and classical forms of type 2 diabetes show impaired expression of genes involved in muscle branched-chain amino acids metabolism
Source: Sci Rep. 2017 Oct 23;7:13850. doi: 10.1038/s41598-017-14120-6 (PMC5653857; doi:10.1038/s41598-017-14120-6)

Early-onset and classical forms of type 2 diabetes show impaired expression of genes involved in muscle branched-chain amino acids metabolism

María Isabel Hernández-Alvarez[1,2,3], Angels Díaz-Ramos[1,2,3], María Berdasco[4], Jeff Cobb[5], Evarist Planet[1], Diane Cooper[7], Agnieszka Pazderska[6], Krzystof Wanic[6], Declan O'Hanlon[6], Antonio Gomez[4], Laura R. de la Ballina[1,2], Manel Esteller[4], Manuel Palacin[1,2,8], Donal J. O'Gorman[7], John J. Nolan[9], and Antonio Zorzano[1,2,3*]

Supplementary data

Supplementary Methods.

Study subjects.

Each subject had medical history taken and underwent physical examination during a screening visit. Routine blood and urine chemistries were analysed. Anthropometric measurements were taken as previously described, including weight (fasting, using a calibrated clinical scales), height (in bare feet, using a stadiometer), BMI, and waist-to-hip ratio (measured around the abdomen at the narrowest point between the iliac crests and the lowest rib margin above the umbilicus, and around the femoral greater trochanters and gluteal mass). This study belongs to a larger study in which patients were subsequently subjected to lifestyle changes. All methods were performed in accordance with the relevant guidelines and regulations.

All patients underwent a 3-hour oral glucose tolerance test (OGTT) after a 12-hour overnight fast. Patients treated with oral hypoglycaemic agents were asked not to take their medications on the day of the test. Individuals who were receiving basal insulin were instructed to omit their dose in the evening before or the morning of the test depending on the individual's regime. 75 grams of glucose, provided by 113 milliliters of Polycal

solution (Nutricia Zoetermeer), diluted with water to up to a total volume of 200 milliliters, was given for the test. The subjects remained in a semi-recumbent position for the duration of the test. Samples for glucose and insulin were drawn and sent to the local lab at 30, 60, 90, 120 and 180 minutes after glucose load.

Muscle biopsies were obtained under local anesthesia from the vastus lateralis muscle (100mg). The muscle samples were immediately frozen in liquid nitrogen and stored for RNA and DNA extraction.

WGCNA

In order to explore gene-gene interactions, we performed a Weighted Co-expression Network Analysis (WGCNA) [1]. The network was constructed based on genes belonging to the most relevant genesets according to results in GSEA. The weighted adjacency matrix was computed using the scale-free topological criterion (beta = 4) and the absolute value of the Pearson correlation as measure of similarity.

Gene modules were identified by hierarchical clustering analysis; for doing so, we used an average linkage as agglomerative method, the Topological Overlap Measure (TOM) as a measure of interconnectedness and the Dynamic Branch Cut [2] method for clustering detection (minimum module size = 10, deep split = 2). Modules were summarized by their corresponding eigengenes. For each gene, strenght of assignment to the corresponding module was measured by the usual Module Membership (MM) metric. For visualization purposes, genes and modules adjacencies were represented in heatmaps based on their TOMs after a power transformation (power = 7). Relationships between modules were further explored using a hierarchical clustering as described above. The resulting modules were tested for enrichment of genes from each of the selected GSEA genesets by means of a exact Fisher's test.

For each gene, association between expression and diabetes was independently assessed using a linear model in an analogous way to that used for differential expression analysis. The t-statistics derived from such models were used as measure of gene significance (GS), in the nomenclature of Network analysis. Association betwen GS and MM and intra-modular connectivity was measured by Pearson and Spearman correlation, respectively. All these analyses were carried out with the WGCNA [3] R package.

Metabolomics.

For absolute quantitation, metabolites were analyzed by isotope dilution UHPLC-MS/MS assays. In brief, 50 µl of EDTA plasma was spiked with stable labeled internal standards and subsequently subjected to protein precipitation by mixing with 200 µl of 1% formic acid in methanol. Following centrifugation, aliquots of clear supernatant were injected onto an Agilent 1290/AB Sciex QTrap 5500 mass spectrometer LC-MS/MS system equipped with a turbo ion-spray source using two different chromatographic systems (mobile phase/column combinations). 3-Methyl-2-oxobutyric acid, 3-methyl-2-oxovalerate, and 4-methyl-2-oxopentanoic acid were eluted with a gradient (mobile phase A: 0.01% formic acid in water; mobile phase B: acetonitrile/methanol 1:1) on a Waters Acquity C-18 BEH column (2.1 mm x 100 mm, 1.7 µm particle size) and detected in negative mode. Isoleucine, leucine, and valine were eluted with a gradient (mobile phase A: 0.05% perfluoropentanoic acid in water; mobile phase B: 0.05% perfluoropentanoic acid in acetonitrile) on a Waters Acquity C-18 BEH column (2.1 mm x 100 mm, 1.7 mm particle size) and detected in positive mode. Quantitation was performed based on the area ratios of analyte and internal standard peaks using a weighted linear least squares regression analysis generated from fortified calibration standards in water, prepared immediately prior to each run. Stable isotope labeled compounds 3-Methyl-2-oxobutyric acid -d_7, 4-methyl-2-oxopentanoic acid -d_3 (used for both 3-methyl-2-oxovalerate and 4-methy-2-oxopentanoic acid), isoleucine-$^{13}C_6$, leucine-d_3, valine-$^{13}C_5$-^{15}N, were used as internal standards.

Muscle Biopsies.

All participants attended in a fasting state for muscle biopsy. Skeletal muscle biopsies were taken from the *m. vastus lateralis* under local anaesthesia, as previously described [4, 5]. An area of skin was anaesthetized with 1% lidocaine and a small (0.5 cm) incision made. The Bergstrom biopsy needle was inserted into the muscle and, with suction applied, approximately 100mg of tissue was extracted. Muscle samples were snap-frozen in liquid nitrogen and stored at −80ºC until RNA and protein extraction.

Mouse models of diabetes.

All animal work was approved and conducted according to guidelines established by the Parc Científic de Barcelona and the University of Barcelona, Spain. All animal procedures complied with the Generalitat of Catalunya guidelines for the Care and Use

of Laboratory Animals and were approved by the Institutional Animal Care and Use Committees on Animal Care. Male *db/db* mice and control wild-type littermates ($n = 8$ per group) were purchased from Charles River Laboratories (Sulzfeld, Germany). Male *ob/ob* mice and C57BL6/J wild-type littermates ($n = 8$ per group) were purchased from Charles River Laboratories (Sulzfeld, Germany), were obtained at the age of 5 weeks. Animal handling was conducted according with the Principles of Laboratory Care and was approved by the Veterinary Inspection Services. Mice were kept in 12hour dark-light periods, and fed standard chow diet and water ad libitum. Animals were killed in a non-fasted state at an age of 8 weeks. Mice were anesthetized using isofluorane and sacrificed by cervical dislocation. Blood, tissues used for RNA, and protein extraction were prepared as reported [6].

Animal plasma and tissue collection

Non-fasted blood was colleted into EDTA-coated tubes via cardiac puncture and centrifugued at 1,200 g at 4C to separate plasma. Plasma and tissues (liver, skeletal muscle and epididymal adipose tissue) were collected, snap-frozen in liquid nitrogen, and stored at -80C.

RNA extraction

RNA isolation was performed using a mix of Trizol and Maxwell ® (Promega). The RNA recovery was around 5mg per sample. All samples showed a very good quality control giving a 10-9 RIN values.

Real-time PCR assays.

Real-time PCR was used for measurement of specific mRNAs (ABI-PRISMA 7700 Sequence Detector, Perkin-Elmer Applied Biosystems, Foster City, CA). All reactions were performed in 384-well MicroAmp Optical plates. Amplification mixes (10 µl) contained the diluted cDNA sample, 2X TaqMan Universal PCR Mastermix or Syber green PCR Mastermix, forward and reverse primers, and probe for the specific mRNAs, as well as Cyclophilin A mix. Thermal cycling conditions included 10 min at 95°C before the onset of the PCR cycles, which consisted of 40 cycles at 95°C for 15 s and 65°C for 1 min. As endogenous control to correct for potential variation in RNA loading and quantification, Human PPIA (cyclophilin A) (#431883E) was used. The probes were supplies as TaqMan reagents kit from Applied Biosystems and used according to the

manufacturer's instructions mRNA expression was calculated using the ΔCT method (Higuchi et al. 1993). Briefly, the ΔCT was calculated by subtracting the CT for cyclophilin A, from the CT for the gene of interest. The relative expression of the gene of interest is calculated using the expression $2^{-\Delta CT}$ and reported as arbitrary units. All PCR runs were performed in duplicate. The sequences for the primer pairs used for human: BCAT2 (Hs01553550_m1), BCKDHA (Hs00958109_m1), BCKDHAB (Hs00609053_m1). For mouse: Mm00802192_m1, Mm00476112_m1, Mm01177077_m1.

Methylation analysis.

BCKDHB DNA methylation analyses. Preliminary DNA analysis of BCKDHB was performed using available data from Infinium HumanMethylation450 BeadChip (Illumina). A three-step normalization procedure was performed using the lumi package available for Bioconductor, within the R statistical environment, consisting of color bias adjustment, background level adjustment and quantile normalization across arrays. Raw intensity DNA methylation files of type 2 diabetic patients from the same cohort than gene expression analysis was determined. Illumina codes for the CpG site studied is cg05170390.

Bisulfite genomic sequencing for BCAT2 promoter. We established BCAT2 CpG island methylation status by PCR analysis of bisulfite-modified genomic DNA, which induces chemical conversion of unmethylated, but not methylated, cytosine to uracil. Briefly, bisulfite modification of 600 ng genomic DNA was carried out with the EZ DNA Methylation Kit (Zymo) following the manufacturer's protocol. After PCR and cloning, ten clones of each mouse sequence and sample were sequenced to determine their degree of methylation. For BCAT2 PCR amplification we used the following set of primers: 5′- TTTTTGTGGTTTTAGAATTAAGG -3′ (sense) and 5′- TATAATCCTCCTCCATAACTCC -3′ (antisense), located at –153 bp and +297 bp from the transcription start site.

Protein extracts from tissues.

Protein homogenate was extracted from approximately 25 mg of tissue. The tissue was immersed in ice-cold medium buffer A (0.1 m KCl, 5 mm MgCl2, 5mm EGTA, 5mm sodium pyrophosphate, pH adjusted to 7.4, 2 µm leupeptin, 2 µm pepstatin, 0.5mm phenylmethylsulphonylfluoride (PMSF) for 2 ×10 min. Following this, the tissue was finely minced in 1/10 (w/v) buffer B (0.25 m sacharose, 50mmKCl, 5 mm EDTA, 1mm sodium pyrophosphate, 5mm MgCl2, pH adjusted to 6.8, 2 µm leupeptin, 2 µm pepstatin, 0.5mm PMSF) and then disrupted with a motor driven Teflon/glass homogenizer. The entire procedure was performed at 0–4◦C. The protein concentration was determined using a Micro BCA protein assay (Pierce, Rockford, IL, USA).

Western blot assays.

Thirty five micrograms of muscle homogenate protein were subjected to SDS–PAGE electrophoresis. Samples were run in a 10% acrylamide/bisacrylamide SDS–PAGE and electroblotted onto nylon filters. These filters were probed with specific antibodies raised against BCAT2 (ab95976 from Abcam), two subunits of the BCKDH system complex (BCKDHA phosphorylated A303-567A and total A303-790A form from Bethyl laboratories; and BCKDHB sc-374630 from Santa Cruz) and β-actin (1/10000, Sigma). Peroxidase-conjugated anti-mouse or anti-rabbit immunoglobulins were used as secondary antibodies (Sigma, USA). The specific proteins were detected by the ECL western blotting detection analysis system (Amersham). The signal obtained with each antibody was quantified on autoradiography films using a laser densitometer.

Statistical analysis.

To measure the association between gene expression and plasma metabolites, Pearson Correlation Coefficients and their corresponding assymptotic 95% confidence intervals and p-values were computed. Correlation coefficients were presented in a symmetric heatmap after clusterization with euclidean distance and Ward agglomeration method.

All these analyses were performed using R (R Core Team (2014). R: A language and environment for statistical computing. R Foundation for Statistical Computing, Vienna, Austria. URL https://www.R-project.org/.) and Bioconductor [7]. R package *hg219.db* was used for probesets annotation (Carlson M. hgu219.db: Affymetrix Human Genome 219 Plate annotation data (chip hgu219). R package version 2.7.1.).

Group comparisons in data other than microarray sample were performed using the Graphpad software. Human data were analysed as unpaired non parametric distribution and Mann-Whitney test was used. For animal studies we assume a normal distribution of the data using an unpaired parametric t-test. Data were presented as mean± standard error unless otherwise stated. Significance was established at $p < 0.05$.

Plasma metabolomics from animal models combined were analyzed by two-way anova and further Bonferroni's multiple comparison test.

References.

[1] Zhang B, Horvath S (2005) A general framework for weighted gene co-expression network analysis. Stat Appl Genet Mol Biol 4: 12

[2] Langfelder P, Zhang B, Horvath S (2008) Defining clusters from a hierarchical cluster tree: the Dynamic Tree Cut package for R. Bioinformatics 24: 719-720

[3] Langfelder P, Horvath S (2008) WGCNA: an R package for weighted correlation network analysis. BMC Bioinformatics 9: 1471-2105

[4] Hernandez-Alvarez MI, Thabit H, Burns N, et al. (2010) Subjects with early-onset type 2 diabetes show defective activation of the skeletal muscle PGC-1{alpha}/Mitofusin-2 regulatory pathway in response to physical activity. Diabetes Care 33: 645-651

[5] Burns N, Finucane FM, Hatunic M, et al. (2007) Early-onset type 2 diabetes in obese white subjects is characterised by a marked defect in beta cell insulin secretion, severe insulin resistance and a lack of response to aerobic exercise training. Diabetologia 50: 1500-1508

[6] Sebastian D, Hernandez-Alvarez MI, Segales J, et al. (2012) Mitofusin 2 (Mfn2) links mitochondrial and endoplasmic reticulum function with insulin signaling and is essential for normal glucose homeostasis. Proceedings of the National Academy of Sciences of the United States of America 109: 5523-5528

[7] Huber W, Carey VJ, Gentleman R, et al. (2015) Orchestrating high-throughput genomic analysis with Bioconductor. Nature methods 12: 115-121.

<u>Supplementary Figures.</u>

Figure S1

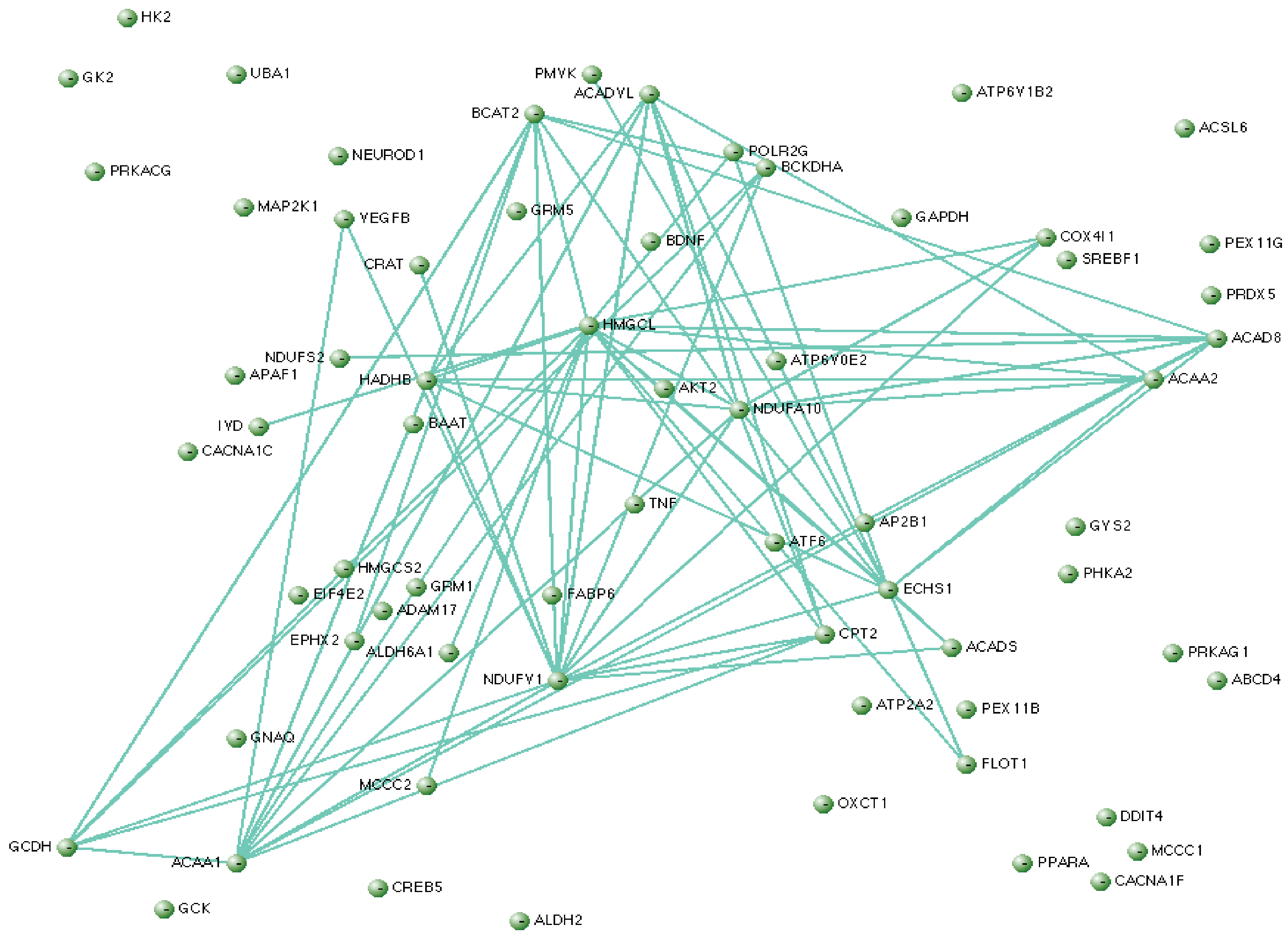
HK2
GK2
UBA1
PMVK
ACADVL
ATP6V1B2
ACSL6
BCAT2
PRKACG
NEUROD1
POLR2G
BCKDHA
MAP2K1
VEGFB
GRM5
GAPDH
COX4I1
PEX11G
BDNF
SREBF1
CRAT
PRDX5
HMGCL
ACAD8
NDUFS2
ATP6V0E2
APAF1
HADHB
ACAA2
AKT2
NDUFA10
IVD
BAAT
CACNA1C
TNF
GYS2
AP2B1
ATF6
HMGCS2
PHKA2
GRM1
EIF4E2
FABP6
ECHS1
ADAM17
EPHX2
CPT2
ALDH6A1
ACADS
PRKAG1
NDUFV1
ABCD4
ATP2A2
PEX11B
GNAQ
FLOT1
MCCC2
OXCT1
DDIT4
GCDH
MCCC1
ACAA1
PPARA
CACNA1F
CREB5
GCK
ALDH2

Supplementary Figure 1. Network of hub genes in the Blue module.

The network of the most highly connected genes in the Blue module. In this network we only display a connection if the corresponding topological overlap is above a threshold of 0.01.

Figure S2

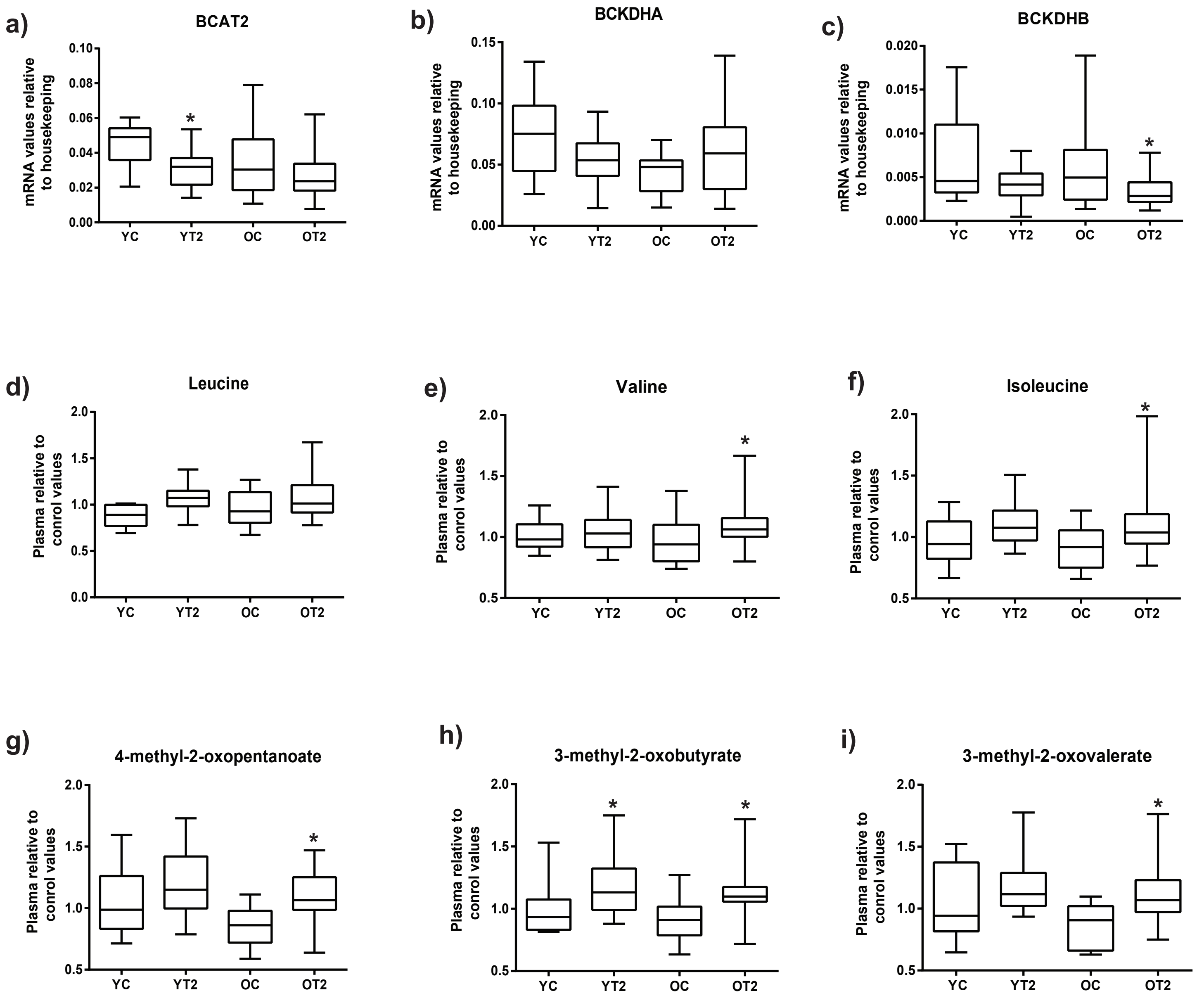
a)
BCAT2
mRNA values relative to housekeeping
0.10
0.08
0.06
0.04
0.02
0.00
YC
YT2
OC
OT2
*
b)
BCKDHA
mRNA values relative to housekeeping
0.15
0.10
0.05
0.00
YC
YT2
OC
OT2
c)
BCKDHB
mRNA values relative to housekeeping
0.020
0.015
0.010
0.005
0.000
YC
YT2
OC
OT2
*
d)
Leucine
Plasma relative to conrol values
2.0
1.5
1.0
0.5
0.0
YC
YT2
OC
OT2
e)
Valine
Plasma relative to conrol values
2.0
1.5
1.0
0.5
YC
YT2
OC
OT2
*
f)
Isoleucine
Plasma relative to conrol values
2.0
1.5
1.0
0.5
YC
YT2
OC
OT2
*
g)
4-methyl-2-oxopentanoate
Plasma relative to conrol values
2.0
1.5
1.0
0.5
YC
YT2
OC
OT2
*
h)
3-methyl-2-oxobutyrate
Plasma relative to conrol values
2.0
1.5
1.0
0.5
YC
YT2
OC
OT2
*
*
i)
3-methyl-2-oxovalerate
Plasma relative to conrol values
2.0
1.5
1.0
0.5
YC
YT2
OC
OT2
*

Supplementary Figure 2. Skeletal muscle gene expression of branched chain amino acids mitochondrial proteins in early-onset type 2 diabetic subjects (YT2) and lateonset type 2 diabetic subjects (OT2) and their respective age-matched control groups (YC and OC).

Real-time PCR was performed in skeletal muscle biopsies: A) BCAT2, B) BCKDHA, and C) BCKDHB); Plasma metabolomics was performed in the same subjects and relative values are shown: D) Leucine, E) Valine, F) Isoleucine and catabolites I) 4-methyl-2-oxopentanoate, J) 3-methyl-2-oxobutyrate and L) 3-methyl-2-oxovalerate. Data are presented as mean ± standard deviation. Statistical analyses comparing type 2 diabetic subjects vs respective controls were performed by unpaired *t*-test $^*p<0.05$.

Figure S3

a)

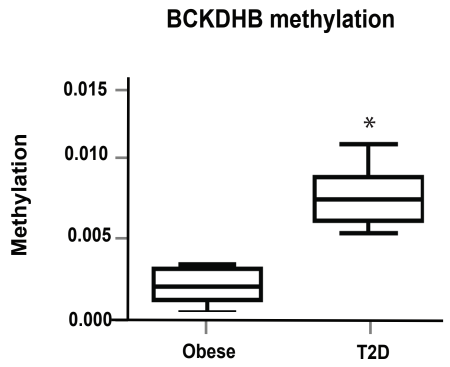
BCKDHB methylation
Methylation
0.015
0.010
0.005
0.000
*
Obese
T2D

Supplementary Figure 3. Effect of type 2 diabetes DNA methylation in skeletal muscle compared to obese nondiabetic states.

Higher methylation of the subunit E1b of the complex BCKDH in skeletal muscle from T2D diabetes subjects respect to obese subjects. Statistical analysis comparing type 2 diabetic subjects vs respective controls was performed by unpaired t-test $^{*}p<0.05$.

Figure S4

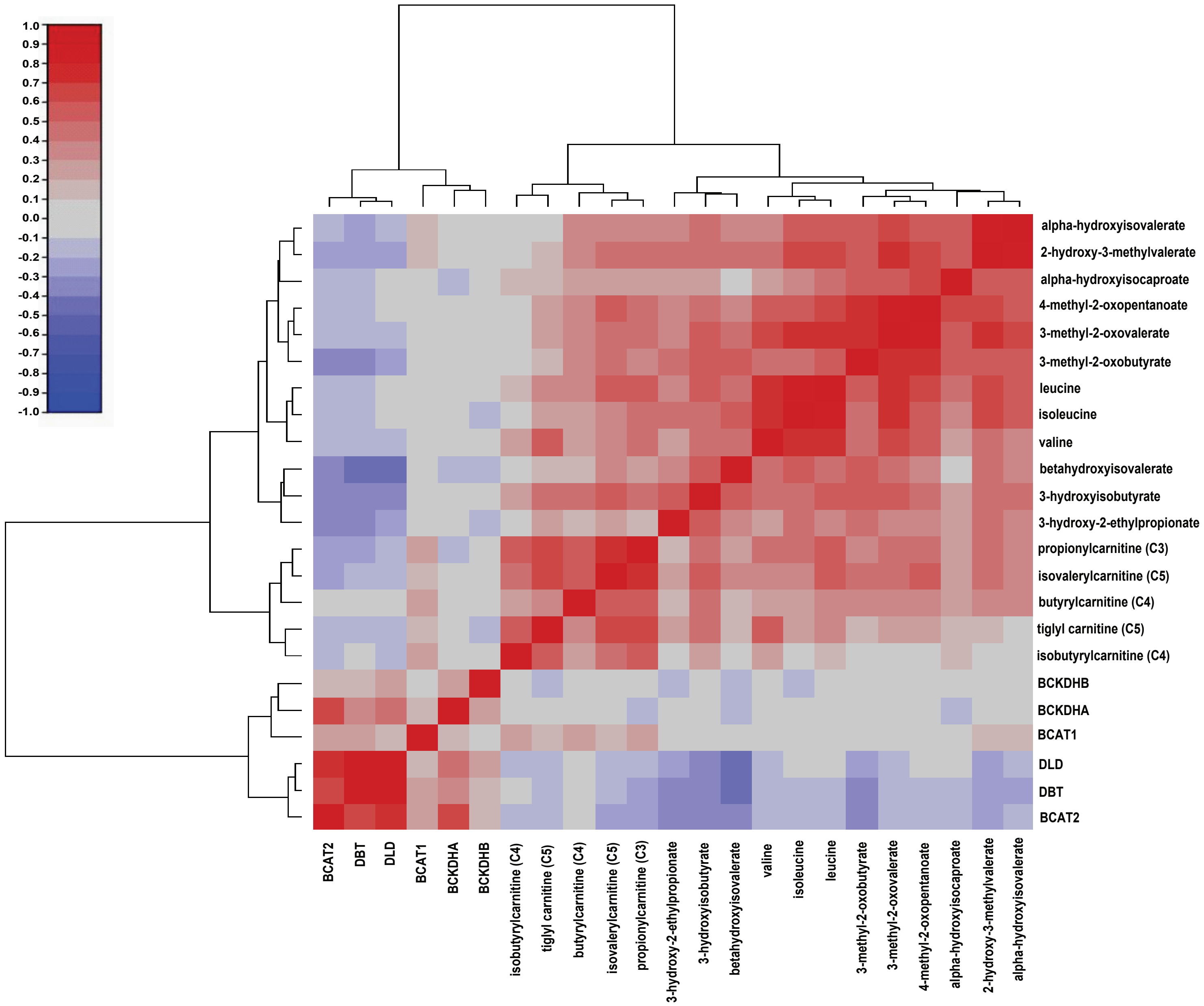

1.0
0.9
0.8
0.7
0.6
0.5
0.4
0.3
0.2
0.1
0.0
-0.1
-0.2
-0.3
-0.4
-0.5
-0.6
-0.7
-0.8
-0.9
-1.0
alpha-hydroxyisovalerate
2-hydroxy-3-methylvalerate
alpha-hydroxyisocaproate
4-methyl-2-oxopentanoate
3-methyl-2-oxovalerate
3-methyl-2-oxobutyrate
leucine
isoleucine
valine
betahydroxyisovalerate
3-hydroxyisobutyrate
3-hydroxy-2-ethylpropionate
propionylcarnitine (C3)
isovalerylcarnitine (C5)
butyrylcarnitine (C4)
tiglyl carnitine (C5)
isobutyrylcarnitine (C4)
BCKDHB
BCKDHA
BCAT1
DLD
DBT
BCAT2
BCAT2
DBT
DLD
BCAT1
BCKDHA
BCKDHB
isobutyrylcarnitine (C4)
tiglyl carnitine (C5)
butyrylcarnitine (C4)
isovalerylcarnitine (C5)
propionylcarnitine (C3)
3-hydroxy-2-ethylpropionate
3-hydroxyisobutyrate
betahydroxyisovalerate
valine
isoleucine
leucine
3-methyl-2-oxobutyrate
3-methyl-2-oxovalerate
4-methyl-2-oxopentanoate
alpha-hydroxyisocaproate
2-hydroxy-3-methylvalerate
alpha-hydroxyisovalerate

Supplementary Figure 4. Plasma metabolomics correlation with skeletal muscle gene expression.

Correlation analysis of plasma branched chain amino acids and their catabolites with gene expression (obtained by RT-PCR) of enzymes involved in branched chain amino acids catabolic pathway in skeletal muscle. Statistics is shown in supplemental table 5.

Figure S5

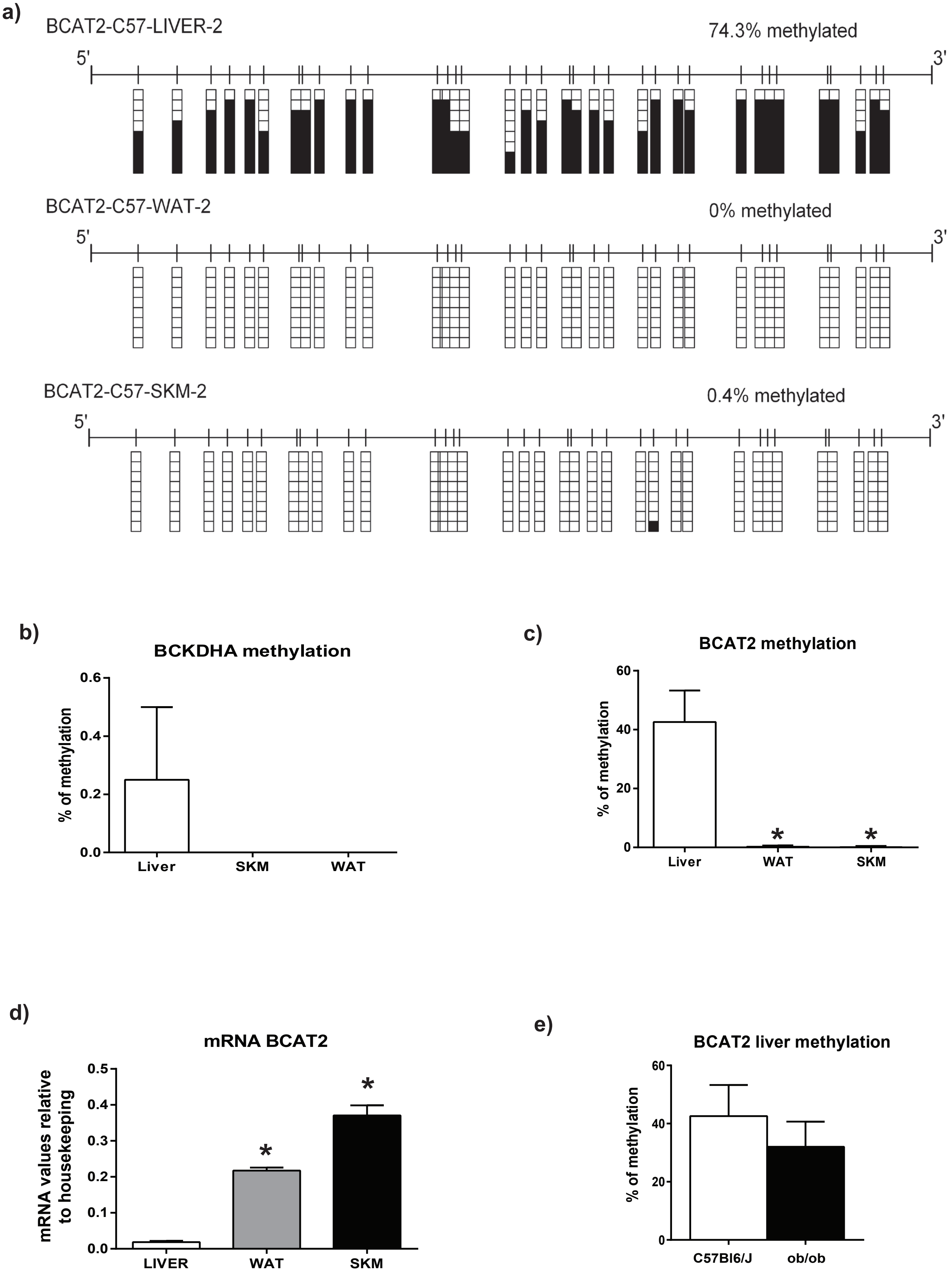

a)
BCAT2-C57-LIVER-2
74.3% methylated
5'
3'
BCAT2-C57-WAT-2
0% methylated
5'
3'
BCAT2-C57-SKM-2
0.4% methylated
5'
3'
b)
BCKDHA methylation
% of methylation
0.6
0.4
0.2
0.0
Liver
SKM
WAT
c)
BCAT2 methylation
% of methylation
60
40
20
0
Liver
WAT
SKM
*
*
d)
mRNA BCAT2
mRNA values relative to housekeeping
0.5
0.4
0.3
0.2
0.1
0.0
LIVER
WAT
SKM
*
*
e)
BCAT2 liver methylation
% of methylation
60
40
20
0
C57Bl6/J
ob/ob

Supplementary Figure 5. Analysis of DNA methylation in mice from genes related to branched chain catabolic pathway.

A) Graphical example of DNA methylation sites in BCAT2 gene in liver, WAT and skeletal muscle from C57BL6 mice. B) Percentage of DNA methylation of BCKDHA gene in liver, skeletal muscle and WAT from C57BL6 mice. C) Percentage of DNA methylation of BCAT2 gene in liver, WAT and skeletal muscle from C57BL6 mice. Data represent mean + SEM of methylation values determined by bisulfite sequencing in eight mice from each category (ten clones per mice and tissue). D) mRNA expression of BCAT2 in liver, WAT and skeletal muscle from C57BL6 mice. E) Percentage of DNA methylation of BCAT2 in lever from C57BL6 and ob/ob mice. Data are means ± SEM. Statistical analyses were performed by unpaired t-test *$p<0.05$.

Figure S6

a)

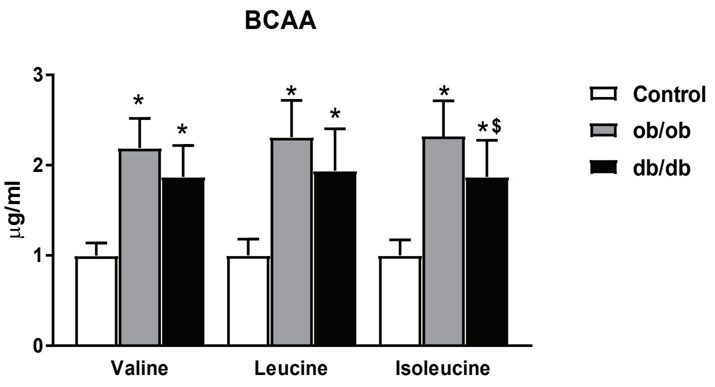

BCAA
μg/ml
0
1
2
3
Valine
Leucine
Isoleucine
Control
ob/ob
db/db
*
*$

b)

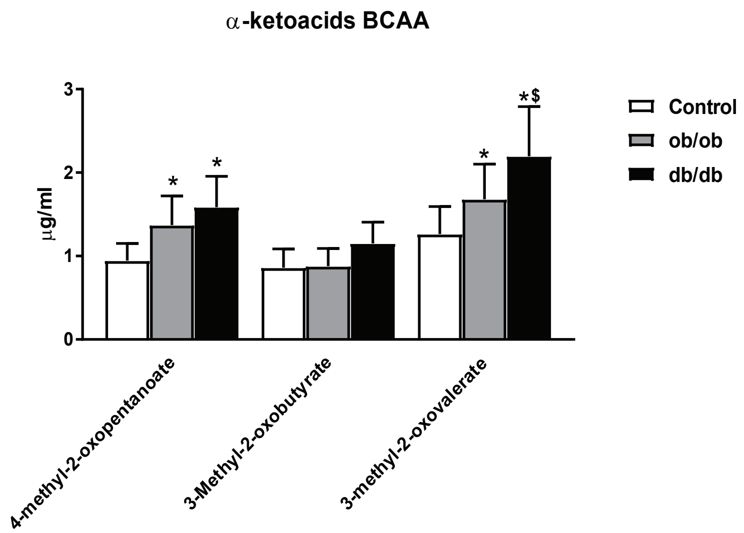

α-ketoacids BCAA
μg/ml
0
1
2
3
4-methyl-2-oxopentanoate
3-Methyl-2-oxobutyrate
3-methyl-2-oxovalerate
Control
ob/ob
db/db
*
*$

Supplementary Figure 6. Plasma metabolites from ob/ob and db/db mice.

A) Grouped plasmatic levels of branched-chain amino acids from mouse models. B) Grouped plasmatic levels of alpha-ketoacids from mouse models. Results were analyzed by two-way Anova and further Bonferroni's multiple comparison test. Data are means ± SD. *$p<0.05$ vs control group. $^{\$}p<0.05$ vs. ob/ob group.

List of Supplementary Tables.

Link:

https://docs.google.com/spreadsheets/d/1B_Yxd1EzQcuBqY8YbHxfLpdrXX9r02ITA5tUwJOMhfM/edit?usp=sharing

Supplementary Table 1.List of probesets differentially expressed in type 2 diabetes.

Supplementary Table 2. Gene sets selected for WCGNA analysis.

Supplementary Table 3. Genes included in the WGCNA Blue module.

Supplementary Table 4. Pairwise correlations and p values between between plasma metabolites and gene expression data obtained by microarrays.

Supplementary Table 5. Pairwise correlations and p values between plasma metabolites and gene expression data obtained by real-time PCR.

Complete gel images from western blots from figures 5 and 6.

Figure 5

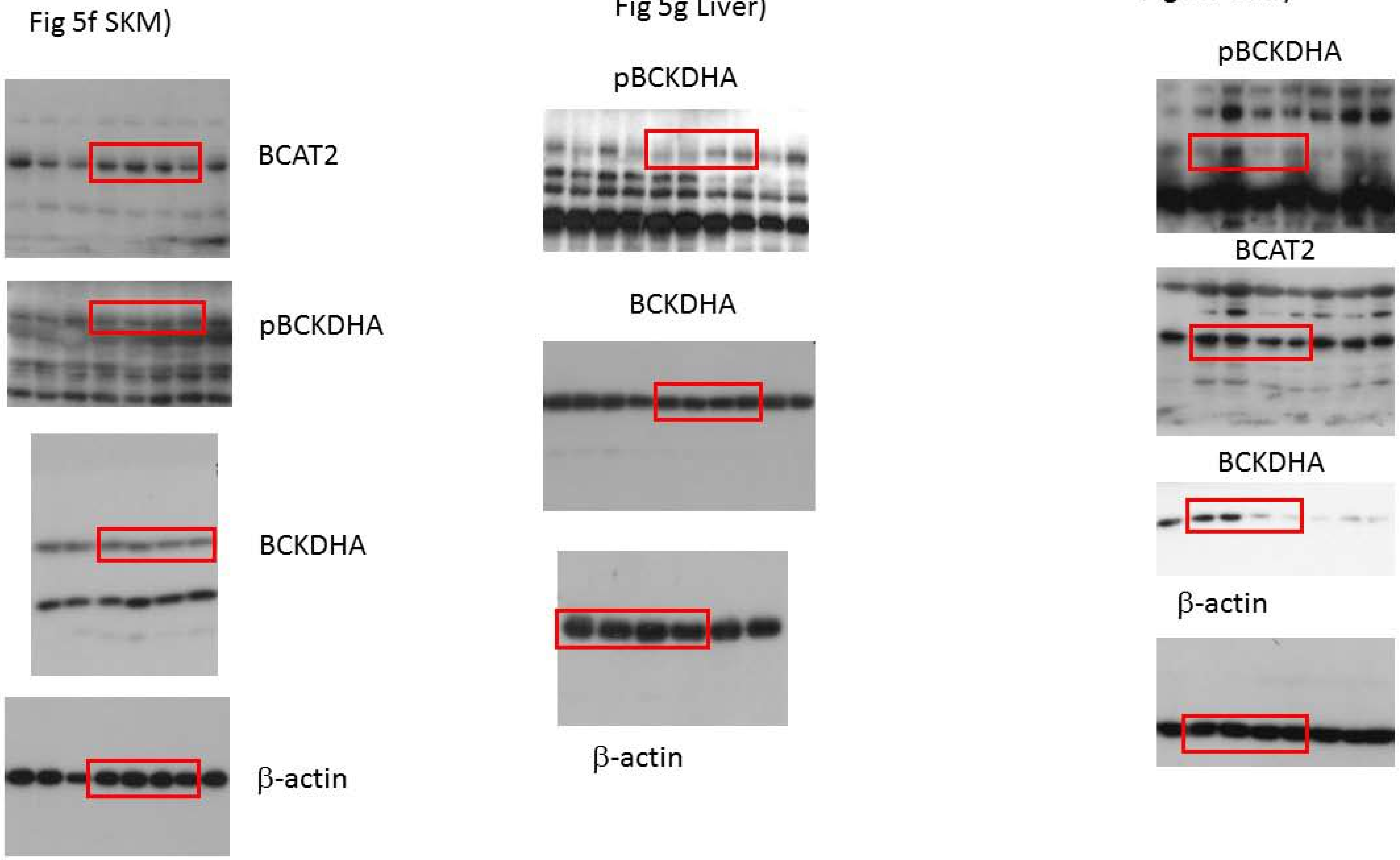

Fig 5f SKM)
BCAT2
pBCKDHA
BCKDHA
β-actin
Fig 5g Liver)
pBCKDHA
BCKDHA
β-actin
Fig 5h WAT)
pBCKDHA
BCAT2
BCKDHA
β-actin

Figure 6

Fig 6f SKM)

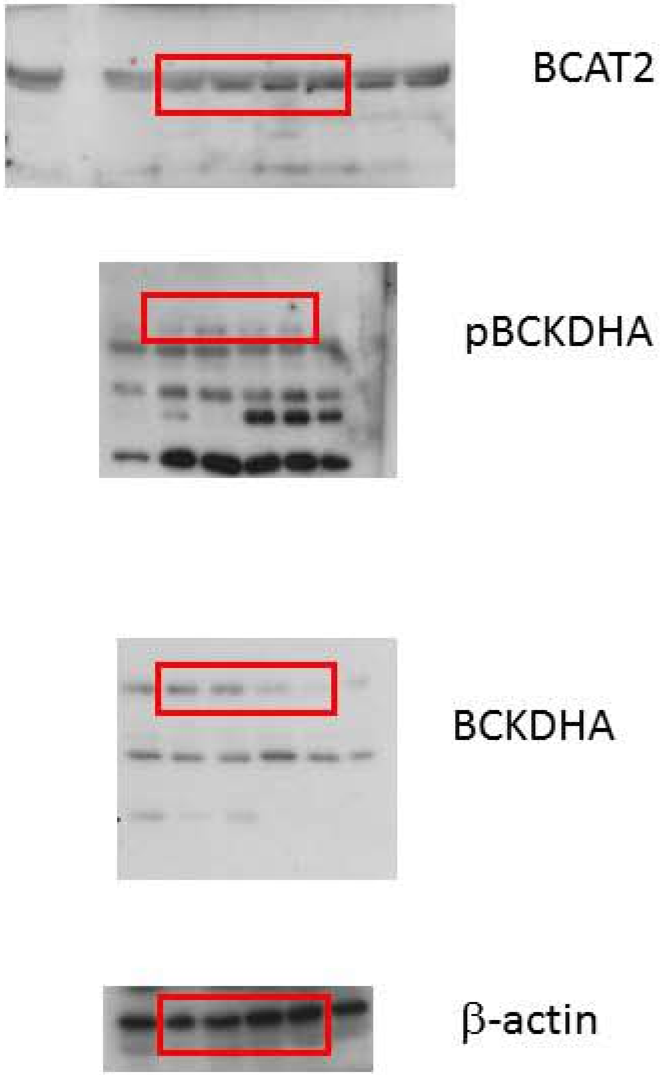

Fig 6g Liver)

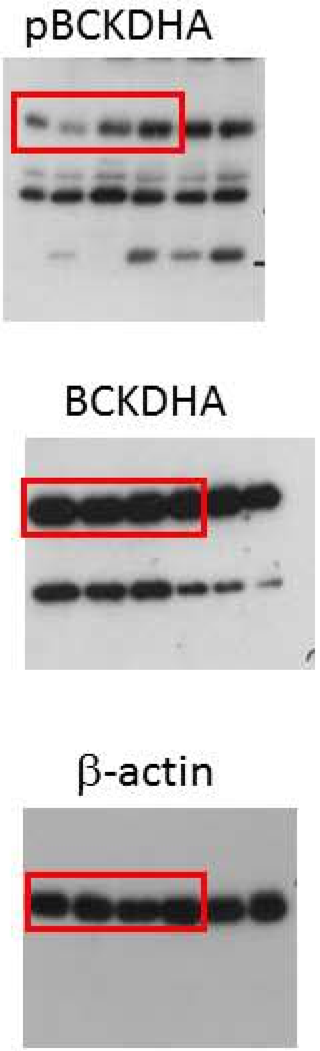

Fig 6h WAT)

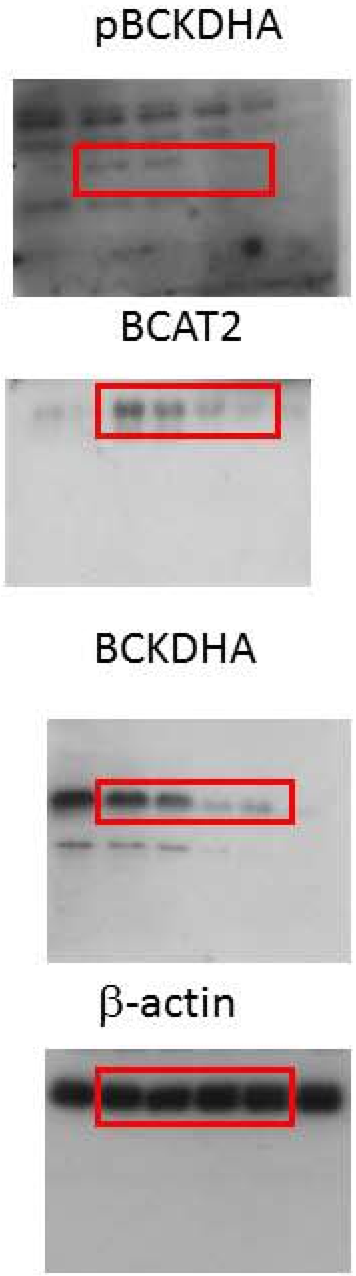

Supplement: Supplementary file 1 — Supplementary data [file 41598_2017_14120_MOESM1_ESM.pdf]
